# Transcriptome-wide N6-methyladenosinem modifications analysis of chicken cecum in responding to *Campylobacter jejuni* inoculation

**DOI:** 10.3389/fimmu.2025.1630008

**Published:** 2025-07-30

**Authors:** Yanan Zhao, Yuanmei Wang, Liying Liu, Yanru Ren, Long Liu, Jiayi Wang, Xianyao Li

**Affiliations:** ^1^ Shandong Provincial Key Laboratory for Livestock Germplasm Innovation & Utilization, College of Animal Science and Technology, Shandong Agricultural University, Tai’an, Shandong, China; ^2^ College of Life Sciences, Shandong Agricultural University, Tai’an, Shandong, China; ^3^ Key Laboratory of Efficient Utilization of Non-grain Feed Resources (Co-construction by Ministry and Province), Ministry of Agriculture and Rural Affairs, Tai’an, Shandong, China

**Keywords:** *Campylobacter jejuni*, chicken, m^6^A, MeRIP-seq, RNA-seq

## Abstract

**Introduction:**

*Campylobacter jejuni (C. jejuni)*, a commensal food-borne pathogen, poses severe threat to human health and poultry industry. N6-methyladenosine (m^6^A) mRNA modification is associated with innate immunity. However, the mechanism of m^6^A modification in *C. jejuni* chicken cecum inoculation remains unclear.

**Methods:**

Here, we characterized the cecal m^6^A modification landscape of chicken in the *C. jejuni*-resistant (R) and susceptible (S) groups using methylated RNA immunoprecipitation sequencing and RNA sequencing (RNA-seq), and further conducted the in vitro *C. jejuni* inflammatory model based on chicken macrophage-like cell line (HD11) to elucidate the specific mechanism.

**Results:**

In the S group, the level of proinflammatory cytokines (IL-8, IL-1β, IL-18, TNF-α, IL-17A) and global RNA methylation were significantly decreased (*P* < 0.05). A total of 30,427 and 30,367 m^6^A peaks were identified in R and S groups, which were primarily located in 3'UTR and CDS regions. Among these, 514 differential m^6^A peaks (270 hypermethylated peaks and 244 hypomethylated peaks) were identified, which mainly correlated with the regulation of canonical NF-kappaB signal transduction, apoptotic signaling pathway, and MyD88-dependent toll-like receptor signaling pathway. Moreover, we identified 365 differentially expressed genes (DEGs), which were mainly associated with regulation of autophagy, and toll-like receptor 9 signaling pathway, intraciliary transport involved in cilium assembly, positive regulation of mTOR signaling, defense response to bacteria. The correlation analysis revealed that m^6^A methylation level correlated positively with gene expression. Further analysis identified 58 differentially methylated genes (DMGs), and mainly involved in apoptosis, autophagy, Notch signaling pathway and defense response to bacteria, which mainly enriched by DMGs including *IFT74, SUSD5, WDR41, STAB2, EPG5* and *FOS*. Furthermore, we found that YTHDC2 could involve in regulating the apoptosis and autophagy process of HD11 cells through altering the expression of DMGs including *IFT74, SUSD5, STAB2, EPG5* and *FOS*, which was confirmed by experiments in vitro.

**Conclusion:**

This result suggested the regulatory role of m^6^A methylation in chicken responds to *C. jejuni* inoculation. Collectively, the current study characterized the m^6^A modification landscape of chicken cecum and identified YTHDC2 acting key regulator responsible for *C. jejuni* inoculation.

## 1 Introduction

*Campylobacter jejuni* (*C. jejuni*), a foodborne bacterial pathogen, is considered a major causative agent of bacterial gastroenteritis (1). It causes severe diarrheal symptoms, accompanied by fever, nausea and abdominal cramping (2), and poses a sever threat to the poultry industry and human health (3). In 2010, *C. jejuni* caused an estimated 96 million cases of diarrheal illness, contributing to approximately 37,600 deaths worldwide (4). Human bacterial gastroenteritis is primarily attributed to *Campylobacter* species, among which *C. jejuni* is responsible for approximately 90% of reported cases (5). Human infection typically results from the ingestion of contaminated animal food products, particularly poultry, where *C. jejuni* colonizes as part of the natural intestinal microbiota (6). *C. jejuni* primarily colonizes the chicken cecum and subsequently disseminates to systemic tissues, resulting in contamination of poultry meat and eggs (7–9). *C. jejuni* can cause intestinal damage, disrupt gut barrier function, and facilitate the translocation of luminal bacteria to internal organs (10, 11). Despite extensive efforts in vaccination and antibiotic applications, *C. jejuni* remains persistent in commercial poultry production. Notably, emerging evidence demonstrates that genetic selection strategies effectively enhance host resistance to *C. jejuni* challenge in chickens, offering a sustainable alternative for disease control (12–14).

The host immune response plays a critical role in responding to *C. jejuni* inoculation. The activation of both innate and adaptive immune responses is critical for controlling *Campylobacter* inoculation (15, 16). Multiple studies have documented immune or metabolic genes closely correlated with resistance in chickens to *C. jejuni* inoculation, including factors such as the major histocompatibility complex (MHC), cadherins, and other genetic elements (17). Briefly, the MHC has been identified as a critical genetic determinant in resistance to *Campylobacter* in commercial broiler chickens (18). The resistance to *C. jejuni* inoculation in the chicken intestine has been linked to a locus spanning *CDH13* (19). Chickens with an inherently high phenotype of pro-inflammatory mediators, including IL-6, CXCLi2, and CCLi2, are more resistant to *Campylobacter* inoculation (20). Disruption of *flhF* abolishes sustained *C. jejuni* invasion capacity in the avian intestinal tract (21). The expression of the host defense peptides (HDPs) including *AvBD1-2*, *CATH1-3*, *AvBD7*, *AvBD4*, and *AvBD6* were suppressed in chicken HD11 cell following *C. jejuni* inoculation (22). Additionally, miR-155, as a vital regulator, could involve in regulating the *C. jejuni* inoculation in chicken (23). The miR-30 and miR-148/miR-152 families exhibit time-dependent regulation in response to *Campylobacter* inoculation in chickens (24). Recent studies revealed that post-transcriptional modifications play crucial roles in regulating the immune system following *C. jejuni* inoculation (25). The recognition of m^6^A methylation within the coding sequence (CDS) by YTHDC2 promotes the overall translation efficiency, whereas knockdown YTHDC2 substantially reduces protein synthesis (26).

N6-methyladenosine (m^6^A), the most prevalent post-transcriptional modification, regulate various biological processes including reproduction (27), growth and development (28, 29), immunity (30, 31), and metabolism (32, 33) through altering mRNA splicing, export, translation, and degradation (34, 35). Recent study revealed that resveratrol augments antioxidant and anti-apoptotic functions in chicken primordial germ cells via m^6^A methylation (36). m^6^A modification is catalyzed by three classes of key regulators, writers (e.g., METTL3, METTL14), erasers (e.g., ALKBH5, FTO) and readers (e.g., YTHDC2, YTHDF2) (37). METTL3 is involved in M1 macrophage polarization and pyroptosis during liver fibrosis (38). It is reported that LPS inoculation alters the m^6^A methylation on the transcripts of GR and impairs its mRNA stability in a YTHDF2-dependent manner, which leads to the decrease of its protein (Zhao et al., 2025). YTHDC2 suppresses antiviral immunity through ISG20-dependent degradation of IFN-β mRNA in macrophages during late-stage viral infection (39). Lactylation of ALKBH5 enhances innate immune responses to DNA viruses including herpesviruses and mpox virus (40). However, the mechanism underlying m^6^A modification in chicken in response to *C. jejuni* inoculation remains poorly understood.

To elucidate the regulatory role of m^6^A modification in the chicken immune response to *C. jejuni* inoculation, the cecal m^6^A modification landscape in susceptible and resistant groups were characterized using MeRIP-seq and RNA-seq. Numerous differential m^6^A methylation peaks and corresponding differentially expressed genes potentially were involved in host defense mechanisms against *C. jejuni*. Notably, YTHDC2 regulates resistance to *C. jejuni* inoculation by modulating immune-related gene expression, as confirmed through *in vitro* experiments. These findings provide new insights into epigenetic regulation of avian host-pathogen interactions.

## 2 Materials and methods

### 2.1 *C. jejuni* inoculation and sample collection

A total of 70 day-3 *C. jejuni*-free specific pathogen-free (SPF) White Leghorn chickens (Jinan SAIS Poultry Co., Ltd, China) were used in the current study. The *C. jejuni* (ATCC 33291) strain was obtained from the China Center of Industrial Culture Collection (CICC). Chickens were raised in sterilized isolators with free access to feed and water. Each chicken was orally inoculated with 0.5 mL of *C. jejuni* solution (1.68 × 10^8^ CFU/mL). All procedures were performed under strict sterilization conditions. At 8 hours post-inoculation with *C. jejuni*, the venous blood, liver, cecal content, and cecum were collected from each individual, and immediately frozen in liquid nitrogen.

To quantify the *C. jejuni* levels, 0.1 g cecal content from each chicken was collected and serially diluted with sterile PBS. Subsequently, the cecum content was cultured on Columbia Blood Agar Base plates (Sigma, USA) under microaerophilic conditions (42°C, 85% N_2_, 10% CO_2_, and 5% O_2_) for 48 hours. Chicken with over 1.86×10^13^ CFU/mL *C. jejuni* in cecal content was classified as the susceptible group (S group), and chicken with below 4.36×10^10^ CFU/mL was assigned to the resistant group (R group). All experimental protocols were approved by the Ethics Committee on the Care and Use of Laboratory Animals at Shandong Agricultural University (Approval Number: SDAUA-2019-060).

### 2.2 Mitochondrial electron microscopy observation

Approximately 1–2 mm^3^ liver tissue from each chicken was fixed in a 2.5% glutaraldehyde and washed with 0.1 M phosphate buffer for three times. After post-fixation with 1% osmium tetroxide in 0.1 M phosphate buffer, tissues were dehydrated through a graded ethanol series (30%, 50%, 70%, 90%, and 100%) for 10 minutes per concentration. The tissue was embedded in epoxy resin (Epon 812, Epon, USA), and polymerized at 60°C for 48 hours. Ultrathin sections (60–90 nm) were prepared and examined using a transmission electron microscope (TEM) (Hitachi, Japan) at a magnifications of 80,000x. ImageJ 1.8.0 was used to quantify the mitochondrial length, width, area, and the number of mitochondrial cristae.

### 2.3 The concentration of immune factors in chicken serum

To collect the serum, the venous blood was collected from each chicken in the R and S groups, and centrifuged at 3000 × g for 10 minutes at 4°C. IgA (ml002792), IL-6 (ml059839), IL-18 (ml042769), IL-1β (ml002787), IL-17A (ml023404), and TNF-α (ml002790) ELISA kits (MLBIO, Shanghai, China) were used to determine the serum cytokines’ levels according to the manufacturer’s instructions, respectively.

### 2.4 m^6^A immunoprecipitation, library construction and sequencing

Total RNA was isolated from cecum sample using the Total RNA Kit I (Omega, Hunt Valley, USA) according to the manufacturer’s instructions. The integrity and concentration of RNA were measured using Agilent 2100 Bioanalyzer (Agilent, California, USA) and Nanodrop 2000 (Nanodrop, Wilmington, DE), respectively. Total RNA was purified using the Dynabeads Oligo (dT) (Thermo Fisher Scientific, Massachusetts, USA), and fragmented into approximately 100 nucleotides. The fragmented RNA of each individual was clustered into two libraries: an immunoprecipitation (IP) library and an input library. For the IP library, the fragmented RNA was incubated with an m^6^A-specific antibody in IP buffer (50 mM Tris-HCl, 750 mM NaCl, and 0.5% Igepal CA-630) for 2 hours at 4°C. The IP and input RNA was then reverse-transcribed into cDNA using SuperScript II Reverse Transcriptase (Invitrogen, Waltham, USA), and second-strand synthesis was conducted with NEBNext^®^ Ultra™ II Directional RNA Library Prep Kit (New England Biolabs, USA). Finally, a total of six libraries in each group (3 replicates × (IP + input)) were constructed, and subjected to paired-end 150 bp sequencing using the Illumina NovaSeq 6000 platform (LC-Bio Technology Co., Ltd., Hangzhou, China).

### 2.5 Bioinformatics analysis

The adapter sequences, duplicate reads, and low quality reads were filtered out from raw data with the default parameters using fastp (41). The clean reads were mapped to the *Gallus gallus* reference genome (GRCg7b) using HISAT2 (42). Peak calling analysis was performed with the R package ExomePeak (43). The candidate peak region within the genome was tested by the Poisson distribution model to assess the statistical significance of read enrichment, and the region with *P* value < 0.05 was considered a peak. The differentially methylated m^6^A peaks (DMPs) between resistant and susceptible groups were identified using Fisher’s test. The distribution of m^6^A peaks across functional elements (5′UTR, start codon, CDS, stop codon, and 3′UTR) was annotated using ANNOVAR (44). Subsequently, the identification and visualization of m^6^A motifs enriched within peak regions were performed using HOMER (45) and the BioSeqUtils package in R (46). The gene expression was quantified using StringTie (47) with default parameters. The differentially expressed genes (DEGs) between R group and S group was identified with DESeq2 package (48). The genes with |log_2_Fold change| ≥ 1 and *P* value < 0.05 were considered DEGs. The DEG harboring at least one DMPs was defined as the differentially methylated gene (DMG). Gene ontology (GO) and Kyoto Encyclopedia of Genes and Genomes (KEGG) enrichment analysis for DMPs, DEGs and DMGs was performed using the LC-Bio OmicStudio platform (https://www.omicstudio.cn/home).

### 2.6 RNA m^6^A dot blot assay

The total RNA from each cecum was denatured at 95°C for 3 minutes and cross-linked to an Immobilon-Ny+ Nylon Membrane Roll (Merck Millipore, Germany). The unbound RNA was washed with Tris-buffered saline containing 0.1% Tween 20 for 5 minutes. After blocking with 5% skimmed milk (BI, Germany) for 1 hour, the membrane was incubated with an anti-m^6^A antibody (1:250 dilution; ab286164, abcam) at 4°C overnight with gentle shaking. Subsequently, the membrane was incubated with an anti-mouse IgG secondary antibody (1:5,000 dilution; ab190475, Abcam) for 1 hour at room temperature. The m^6^A levels were visualized using a chemiluminescent substrate in a chemiluminescence imaging system (Fusion Fx Vilber Lourmat, France) and then quantified using ImageJ 1.8.0.

### 2.7 MeRIP-PCR

Following RNA extraction, poly(A)+ RNA was selectively purified using oligo(dT) magnetic beads (Thermo Fisher Scientific, Massachusetts, USA), and fragmented into approximately 100 nts using the Magnesium RNA Fragmentation Kit (New England Biolabs, USA) following the manufacturer’s instructions. The fragmented RNA was then subjected to immunoprecipitation with an m^6^A-specific antibody (Synaptic Systems, Göttingen, Germany) conjugated to Protein A/G magnetic beads (Invitrogen, USA). Both the immunoprecipitated RNA and input RNA were reverse transcribed into cDNA using the PrimeScript RT Reagent Kit (Takara, Dalian, China). qRT-PCR was performed using SYBR Premix Dimer Eraser (Takara, Dalian, China) and specific primers (Sangon, Shanghai, China) on Roche LightCycler^®^ 96 System (**Supplementary Table S1**).

### 2.8 Cell culture, siRNA and LPS challenge

The chicken macrophage-like cell line (HD11) was provided by ShanghaiNulen Biotech. (Shanghai, China). HD11 were cultured in Dulbecco’s modified Eagle’s medium (DMEM) (Gibco, Invitrogen, Carlsbad, CA), supplemented with 10% fetal bovine serum (Gibico, Thermo Fisher Scientific, Australia) and 1% penicillin and streptomycin (Servicebio, Beijing, China) at 37 °C in a humidified atmosphere of 5 % CO_2_ for amplification. Cells were subcultured when they reached 80% to 90% confluence.

The small interfering RNA (si-YTHDC2) for YTHDC2 (Sense: 5’-CAGCUUUAAUUGUGAGAAATT-3’; Anti-sense: 3’-UUUCUCACAAUUAAAGCUGTT-5’) and negative control si-NC were obtained from Sangon Biotech (Shanghai, China). *C. jejuni* lipopolysacharide (LPS) was obtained from FUJIFILM Wako (Cat. No. 128-05671, Japan). The HD11 cells were seeded in 6-well plates at a density of 1 × 10^6^ cells per well and cultured for 24 h. The si-YTHDC2 or si-NC was transfected using Lipofectamine™ 3000 (Thermo Scientific, Invitrogen, US) in serum-free Opti-MEM^®^I Medium (Gibco, Invitrogen, Carlsbad, CA), and incubated at 37 °C in a humidified atmosphere of 5 % CO_2_ for 8 h. Following 24 hours transfection, the cells were incubated with LPS challenge (5 μg/mL) for 8 h, and the expression of YTHDC2 was detected using RT-qPCR (**Supplementary Figure S1**).

### 2.9 Flow cytometric analysis

Apoptosis assay was performed using the Annexin V-FITC/propidium iodide Cell Apoptosis Detection Kit (Servicebio, Wuhan, China) followed by flow cytometry analysis. Briefly, the cells were rinsed and resuspended with 1× binding buffer to a concentration of 5 × 10^6^ cells/mL. A 100 μL cell suspension was incubated with Annexin-FITC (5 μL) and propidium iodide (5 μL) for 10 min at room temperature in the dark. Data were analyzed using Flowjo software (Version: 10.9.1).

### 2.10 Quantitative real-time polymerase chain reaction

Total RNA from cecum or HD11 was extracted using TRIzol Reagent (Thermo Scientific, Invitrogen, US) following the manufactures’ instructions. One μg total RNA from each sample used for RNA sequencing was reverse-transcribed into cDNA with PrimeScript™ RT Reagent Kit (Takara, Japan). Quantitative real-time PCR (qRT-PCR) was performed using SYBR Premix Dimer Eraser (Takara, Japan) and gene-specific primers (Sangon, Shanghai, China) (**Supplementary Table S1**). The relative gene expression level was calculated using the 2^ -ΔΔCt^ method.

### 2.11 Statistical analysis

In the current study, the statistical analysis was performed with SPSS 26.0 software. T-tests and One-way analysis of variance (ANOVA) followed by Tukey’s *post hoc* test were employed to ascertain differences among groups. Among these, T-tests were used for two groups and One-way ANOVA with Tukey’s multiple comparisons were used for multiple groups (n = 4). The data were presented as mean ± SEM. Results with *P* value < 0.05 were considered statistically significant.

## 3 Results

### 3.1 The characterization of immune related traits of chicken in the susceptible and resistant groups

**Figure 1:**
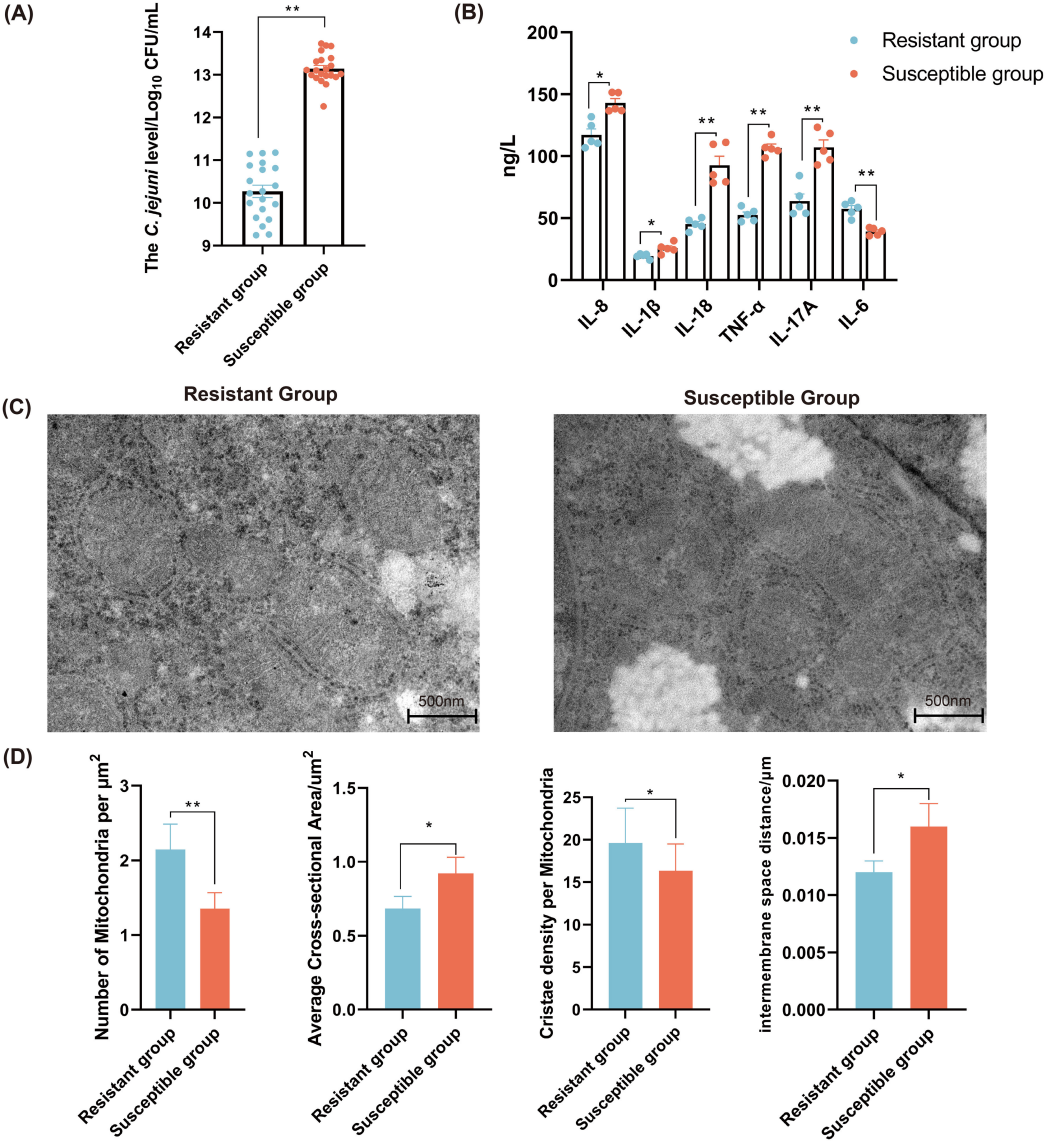
The characterization of immune related traits of chicken in the *C. jejuni*-susceptible (S) and resistant (R) groups. **(A)** The *C. jejuni* colonization levels in chicken cecum of resistant and susceptible groups. **(B)** The levels of cytokines in chicken serum in the *C. jejuni*-susceptible and resistant groups. Scale bar, 500 nm. **(C)** The mitochondrial morphology and ultrastructure of chicken liver in the *C. jejuni*-susceptible and resistant groups. **(D)** The indexes of mitochondria of liver in the *C. jejuni*-susceptible and resistant groups. The data are pooled from 2 independent experiments with 5 replicates per group (n = 5) and presented as the mean ± SEM; *, ** representing *P* < 0.05, and *P* < 0.01, respectively.

The *C. jejuni* colonization level in chicken cecum of the R group was significantly lower than that of the S group (*P* < 0.01) (**Figure 1A**). The level of proinflammatory cytokine (IL-8, IL-1β, IL-18, TNF-α, and IL-17A) in the S group was significantly higher compared to the R group. Whereas, the level IL-6 was significantly decreased in the S group (*P* < 0.05) (**Figure 1B**). The mitochondria in the R group were closely arranged, clearly visible, and evenly distributed, whereas those in the S group appeared damaged and swollen (**Figure 1C**). Additionally, the average cross-sectional area and the inter-membrane space distance of mitochondria in the R group were significantly smaller than those in the S group (*P* < 0.05). Whereas, the number of mitochondria per μm^2^ and the cristae density per mitochondria in the R group were significantly higher than those in the S group (**Figure 1D**).

### 3.2 Characterization of m^6^A methylation

**Figure 2:**
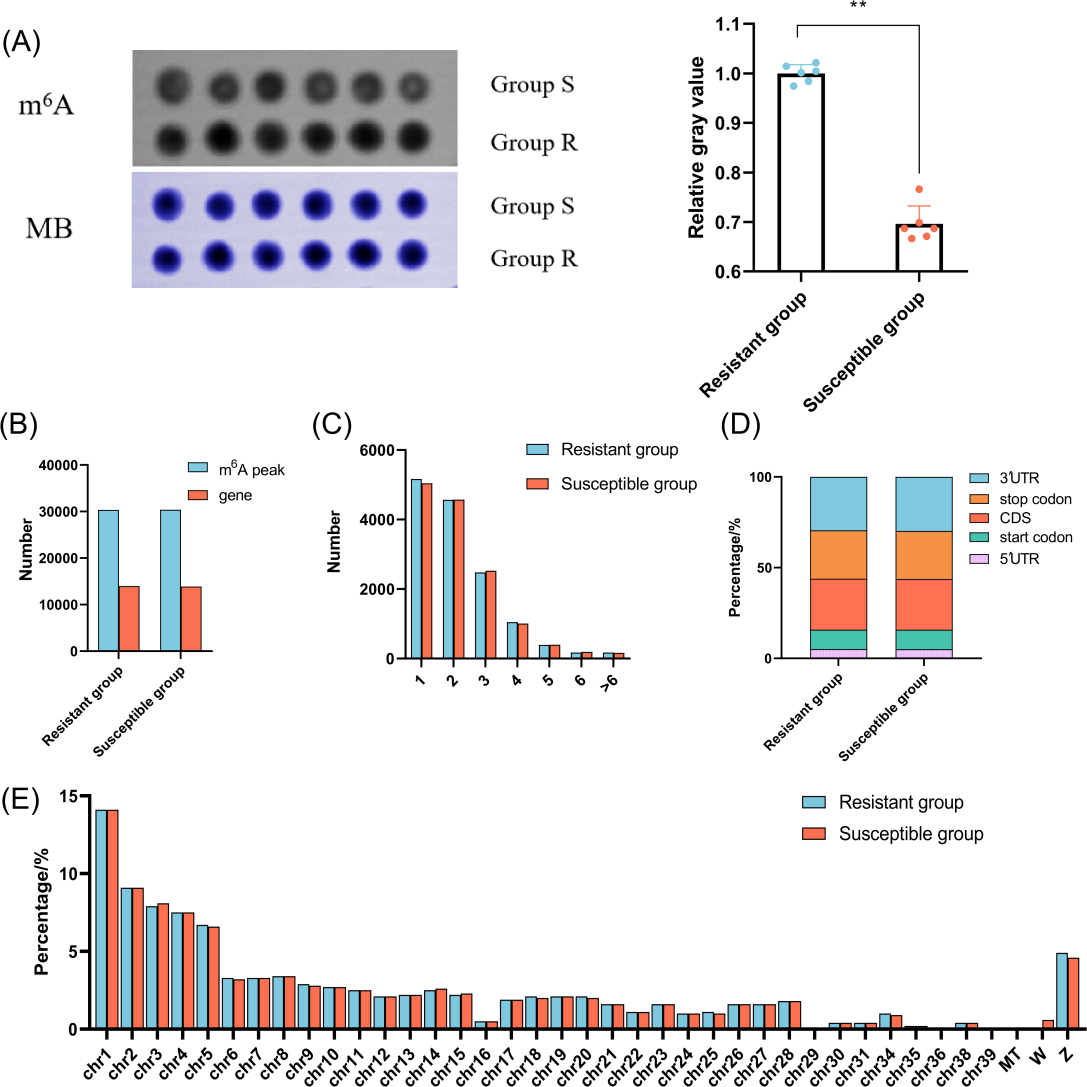
The characterization of the m^6^A methylation of chicken cecum in the *C. jejuni*-susceptible (S) and resistant (R) groups. **(A)** The global m^6^A levels of chicken cecum in the *C. jejuni*-susceptible and resistant groups. The data are pooled from 2 independent experiments with 6 replicates per group (n = 6) and presented as the mean ± SEM; ** representing *P* < 0.01. **(B)** The number of identified m^6^A peaks and genes in the *C. jejuni*-susceptible and resistant groups. **(C)**. The number of peaks in the corresponding genes in the *C. jejuni*-susceptible and resistant groups. **(D)** The distribution of m^6^A peaks in different genomic features in the *C. jejuni*-susceptible and resistant groups. **(E)** The density of m^6^A peaks on chromosomes in the *C. jejuni*-susceptible and resistant groups.

To elucidate the mechanism underlying chicken responding to *C. jejuni* inoculation, we evaluated the global level of m^6^A in chicken cecum in the R and S groups using dot blot method. As shown in **Figure 2A**, the global level of m^6^A modification decreased substantially in S group (*P* < 0.01). Further analysis of MeRIP-seq and RNA-seq indicated that 40–50 million raw reads were obtained from each sample. After removing the low quality reads, more than 39 million clean reads with Q20 values above 98% were generated from each sample (**Supplementary Table S2**), and more than 86.86% clean reads could be uniquely aligned to the chicken genome. Moreover, more than 91.82% clean reads could be mapped to the exon region (**Supplementary Figure S2**). A total of 30,427 and 30,367 m^6^A peaks, corresponding to 13,969 and 13,875 genes, were identified in the R and S groups, respectively (**Figure 2B**). Most genes contained just 1–2 peaks (**Figure 2C**). The m^6^A peaks in the R and S groups were predominantly enriched in the 3′UTR, CDS region, and stop codon, followed by the start codon and 5′UTR (**Figure 2D**). Moreover, m^6^A modifications were widely distributed throughout the chicken genome, with the highest number of peaks located on chromosome 1, accounting for 14.1% in each group (**Figure 2E**). Additionally, enriched motifs in both groups matched the canonical m^6^A motifs ‘RRACH’ and ‘DRACH’ (R = A or G; D = A, G or U; H = A, C or U), while multiple motifs corresponding to non-canonical m^6^A sites including ‘CUACG’ and ‘CGACG’ were also identified (**Supplementary Figure S3**).

### 3.3 Identification and functional analysis of DMPs

**Figure 3:**
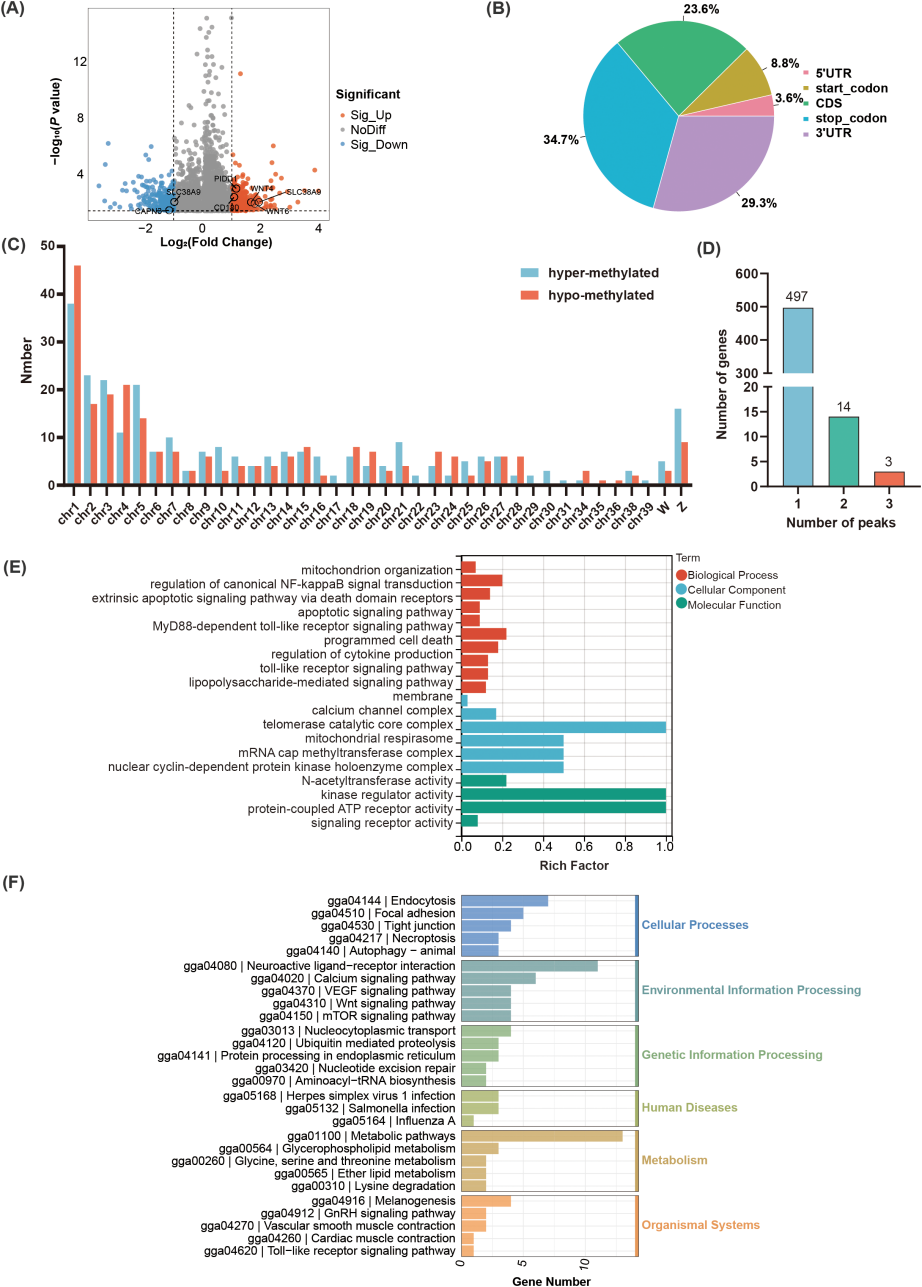
Identification and functional analysis of the identified m^6^A peaks in the *C. jejuni*-susceptible (S) and resistant (R) groups. **(A)** The identified m^6^A peaks in *C. jejuni*-susceptible and resistant groups. **(B)**. The distribution of differential m^6^A peaks in different genomic features in the resistant and susceptible groups. **(C)**. The density of m^6^A peaks on chromosomes. **(D)** The number of differential m^6^A peaks in the corresponding genes. **(E)** The enriched GO terms for the differential m^6^A peaks. **(F)** The enriched KEGG pathway of differential m^6^A peaks.

There were 514 DMPs identified between the R group and the S group including 270 hyper-methylated and 244 hypo-methylated peaks (*P* < 0.05, |log_2_ Fold change| ≥ 1) (**Figure 3A**, **Supplementary Table S3**), which mainly located in the stop codon (34.7%) (**Figure 3B**). Among which, the 270 hyper-methylated peaks were widely distributed on 36 chromosomes, while the 244 hypo-methylated peaks were widely distributed on 32 chromosomes (**Figure 3C**). Moreover, there were 38 hyper-methylated and 46 hypo-methylated peaks mainly located on chromosome 1. These DMPs were annotated to 514 genes, approximately 98% genes contained one m^6^A peak (**Figure 3D**). GO enrichment analysis indicated that the DMPs were significantly enriched in 324 terms (212 biological process (BP), 76 molecular function (MF), 36 cellular component (CC)) (*P* < 0.05) (**Supplementary Table S4**). In terms of BP, the major immune-related pathways including regulation of mitochondrion organization, regulation of canonical NF-kappaB signal transduction, apoptotic signaling pathway, MyD88-dependent toll-like receptor signaling pathway, and lipopolysaccharide-mediated signaling pathway were significantly enriched (**Figure 3E**). Notably, *PIDD1*, *ZFAND6*, and *CAPN3* were significantly enriched in the regulation of canonical NF-kappaB signal transduction, and apoptotic signaling pathway. *PTAFR* and *CD180* were significantly enriched in the lipopolysaccharide-mediated signaling pathway. KEGG pathway enrichment results revealed that the DMPs were significantly enriched in Wnt signaling pathway, mTOR signaling pathway, Toll-like receptor signaling pathway, calcium signaling pathway, VEGF signaling pathway, autophagy, and tight junction (*P* < 0.05) (**Figure 3F**). Notably, *WNT6*, *PRKCB*, *WNT4*, and *SLC38A9* were demonstrated significantly associated with the mTOR signaling pathway.

### 3.4 Correlated analysis of m^6^A modification and gene expression

**Figure 4:**
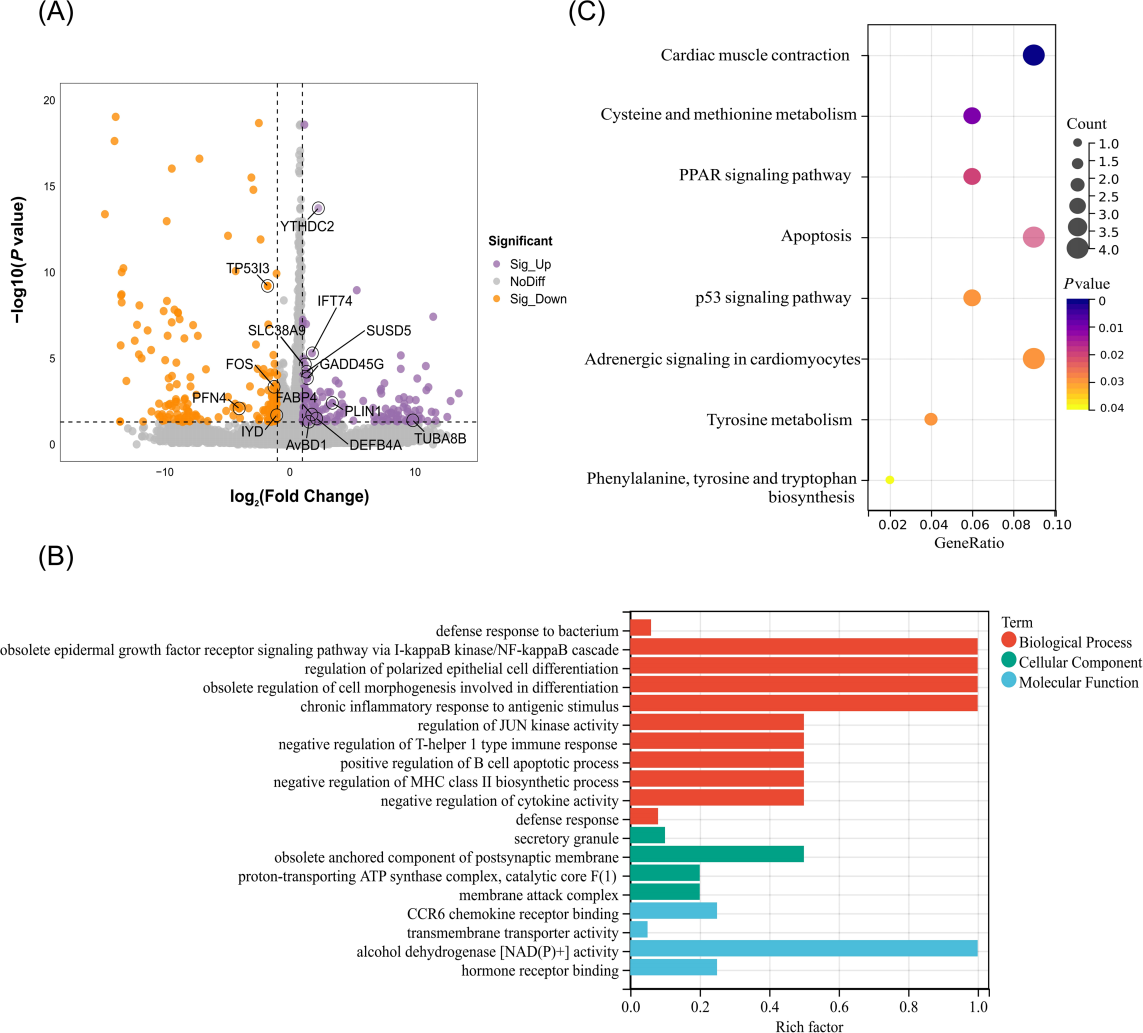
Identification and functional analysis of differentially expressed genes in the *C. jejuni*-susceptible (S) and resistant (R) groups. **(A)** The identified genes in the *C. jejuni*-susceptible and resistant groups. **(B)** The enriched GO terms of differentially expressed genes. **(C)** The enriched KEGG pathway of differentially expressed genes.

To elucidate the functional consequences of the gene expression modified by m^6^A methylation, the global transcriptomic landscape of chicken cecum in the R and S groups were performed using RNA-seq. Totally, 365 DEGs were identified including 166 upregulated genes and 199 downregulated genes (*P* < 0.05, |log_2_Fold change| ≥ 1) (**Figure 4A**, **Supplementary Table S5**). Notably, the expression of m^6^A modification related gene *YTHDC2* were upregulated in the R group, but no significance was observed in other m^6^A modification related genes such as *METTL14*, *FTO*, *ALKBH5* (**Supplementary Figure S4**). Further GO and KEGG analysis for DEGs identified 193 significantly enriched terms (128 BP, 49 MF, 16 CC) (*P* < 0.05) (**Supplementary Table S6**). In terms of BP, the major immune-related terms including defense response to bacterium, regulation of polarized epithelial cell differentiation, negative regulation of T-helper 1 type immune response, positive regulation of B cell apoptotic process, and negative regulation of cytokine activity were significantly enriched (**Figure 4B**). Among these, *IL-10*, *DEFB4A*, and *AvBD1* were significantly enriched in defense response to bacterium. KEGG enrichment results showed that the DEGs were significantly enriched in eight pathways including PPAR signaling pathway, apoptosis, p53 signaling pathway, tyrosine metabolism (*P* < 0.05) (**Figure 4C**).

**Figure 5:**
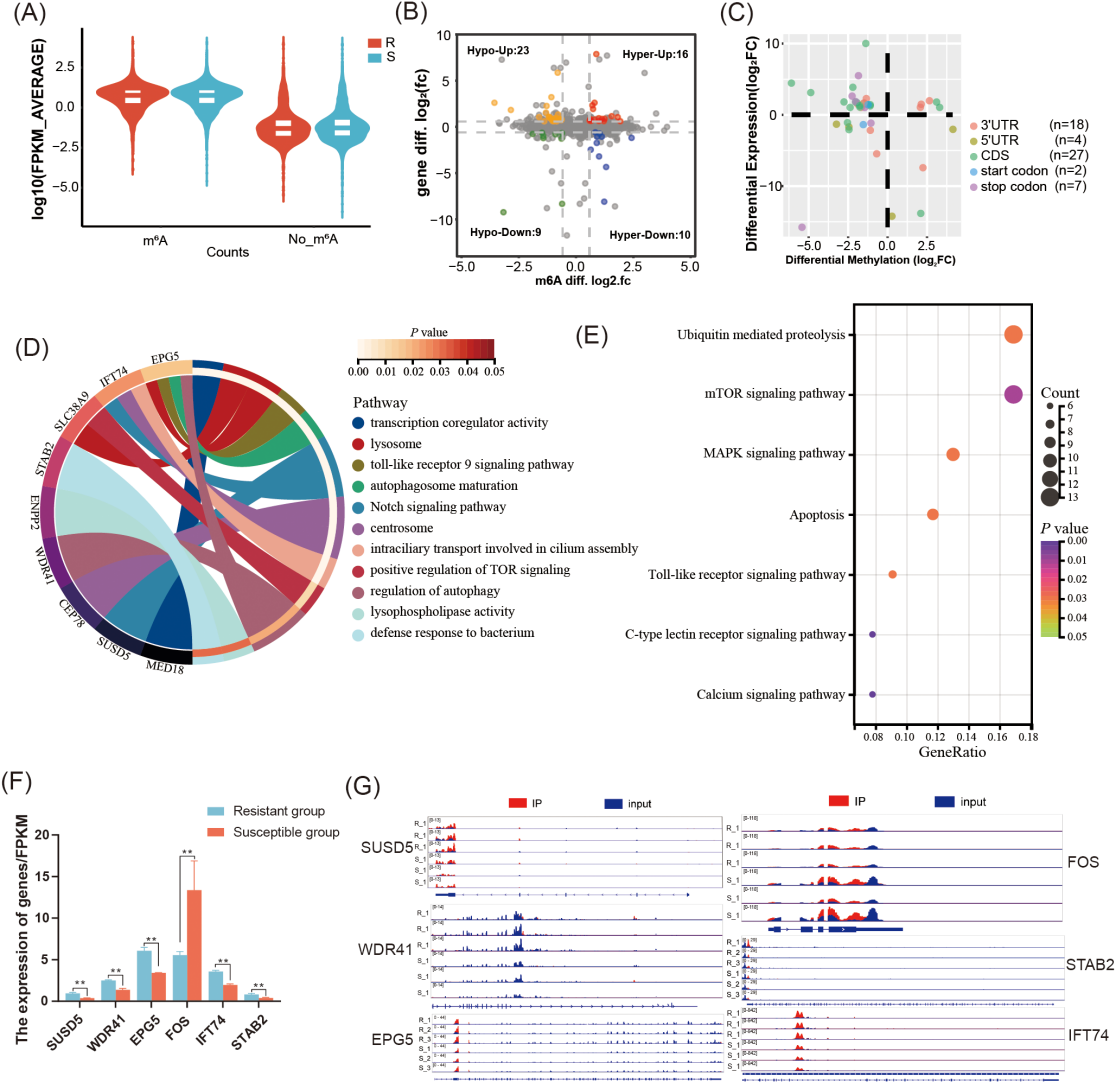
Integrated analysis of MeRIP-seq and RNA-seq in the *C. jejuni*-susceptible (S) and resistant (R) groups. **(A)** The gene expression of genes with or without m^6^A modifications in the resistant and susceptible groups. **(B)** Distribution of genes with significant changes in both gene expression levels as well as m^6^A levels (Hyper-up: m^6^A levels upregulated and mRNA expression upregulated; Hyper-down: m^6^A levels upregulated as well as mRNA expression downregulated; Hypo-up: m^6^A levels downregulated as well as mRNA expression upregulated; Hypo-down: m^6^A levels downregulated as well as mRNA expression downregulated. **(C)** The distribution of differentially expressed genes containing differential m^6^A peaks in different genomic features. **(D)** The enriched GO terms of differentially expressed genes containing differential m^6^A peaks **(E)** The enriched KEGG pathway of DMGs with differential peaks. **(F)** The expression of differentially expressed genes containing differential m^6^A peaks *SUSD5*, *WDR41*, *EPG5*, *FOS*, *STAB2*, and *IFT74* in the resistant and susceptible groups. The data are pooled from 2 independent experiments with 6 replicates per group (n = 6) and presented as the mean ± SEM; ** representing *P* < 0.01. **(G)** The distribution m^6^A peaks located in *SUSD5*, *WDR41*, *EPG5*, *FOS*, *STAB2*, and *IFT74*.

To elucidate the regulatory role of m^6^A modification, the correlation between m^6^A modification and gene expression was performed. In general, we found that the expression of gene containing m^6^A peak was significantly higher than that of genes without m^6^A modifications in both the R and S groups (*P* < 0.05) (**Figure 5A**). Further analysis revealed that 58 DMGs were identified in R group including 26 hyper-methylated DMGs (16 mRNAs upregulated and 10 mRNAs downregulated), and 32 hypo-methylated DMGs (23 mRNAs upregulated and 9 mRNAs downregulated) (**Figure 5B**, **Supplementary Table S7**). The m^6^A peaks of the DMGs predominantly located in CDS region (n=27) and 3’UTR (n=18) (**Figure 5C**). The GO enrichment results showed that these DMGs were mainly enriched in 11 terms including autophagy, toll-like receptor 9 signaling pathway, Notch signaling pathway, intraciliary transport involved in cilium assembly (*P* < 0.05) (**Figure 5D**). KEGG enrichment results showed that these DMGs were significantly enriched in 7 pathways including C-type lectin receptor signaling pathway, Calcium signaling pathway, toll-like receptor signaling pathway, apoptosis, MAPK signaling pathway, and mTOR signaling pathway (*P* < 0.05) (**Figure 5E**). Interestingly, the hypo-methylated DMGs including *SUSD5* and *IFT74* were significantly enriched in Notch signaling pathway, but the expression at mRNA level was increased. The regulation of autophagy pathway was enriched by two hyper-methylated DMGs *WDR41* and *EPG5*, which have higher mRNA expression. Moreover, the DMG *FOS* with hypo-methylated level and lower expression at mRNA level was significantly enriched in apoptosis pathway. The hypo-methylated DMG *STAB2* was mainly associated with defense response to bacteria (**Figures 5F, G**).

### 3.5 The regulatory role of YTHDC2 in HD11 cells responding to *C. jejuni* colonization

**Figure 6:**
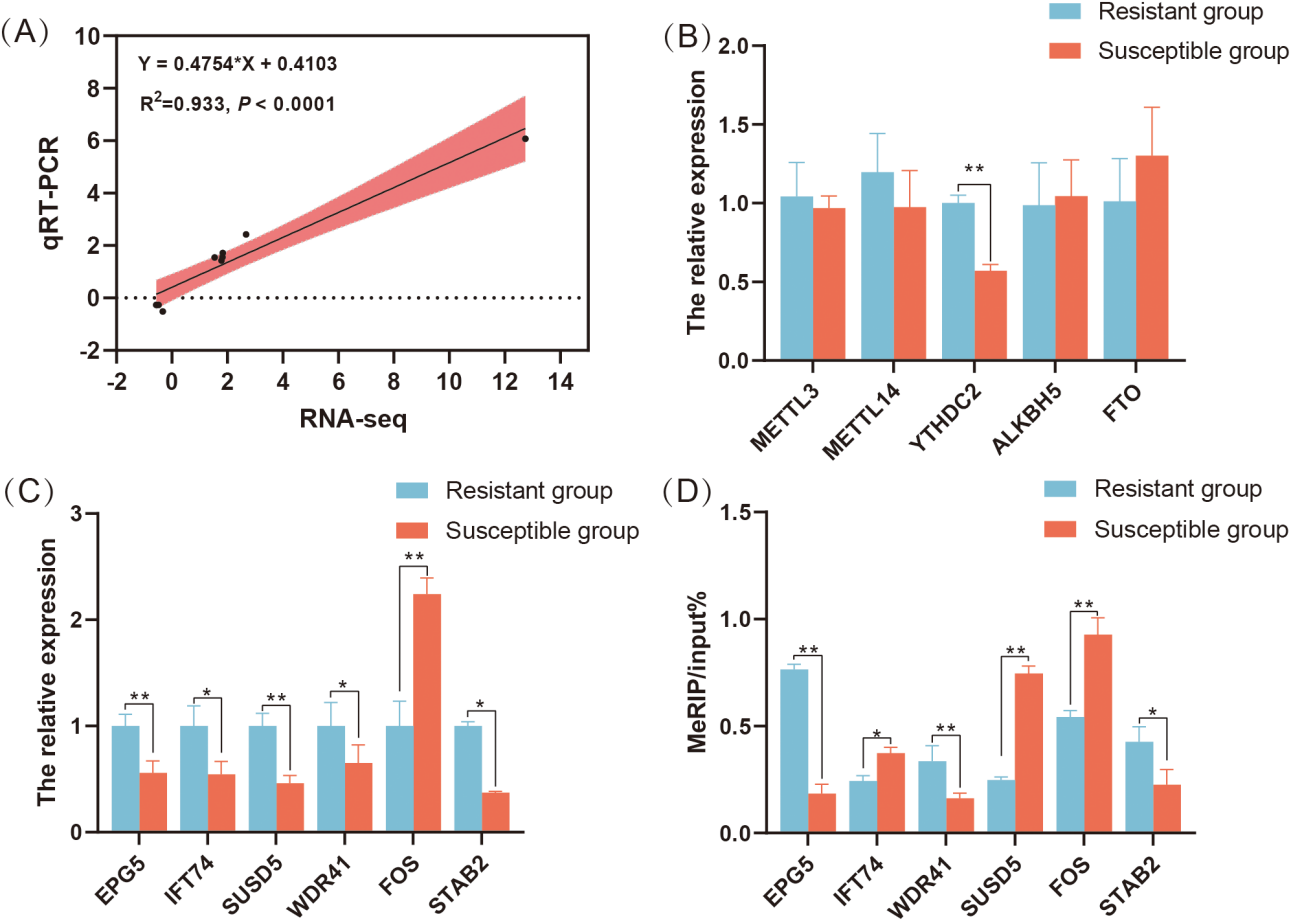
Validation of m^6^A peaks and mRNA levels of differentially expressed genes containing differential m^6^A peaks in the *C. jejuni*-susceptible (S) and resistant (R) groups. **(A)** qRT-PCR validation of gene expression in the resistant and susceptible groups. **(B)** The expression levels of m^6^A modification related genes *METTL3*, *METTL14*, *YTHDC2*, *ALKBH5*, and *FTO* in the resistant and susceptible groups of chicken cecum. **(C)** qRT-PCR results of *SUSD5*, *WDR41*, *EPG5*, *FOS*, *STAB2*, and *IFT74* in the resistant and susceptible groups of chicken cecum. **(D)** meRIP-qPCR results of *SUSD5*, *WDR41*, *EPG5*, *FOS*, *STAB2*, and *IFT74* in the resistant and susceptible groups of chicken cecum. The data are pooled from 2 independent experiments with 6 replicates per group (n = 6) and presented as the mean ± SEM; * and ** represent *P* < 0.05, *P* < 0.01, respectively.

In general, the qRT-PCR results for seven randomly selected genes were highly correlated with the sequencing results (R^2^ = 0.933, *P* < 0.0001) (**Figure 6A**). Additionally, the m^6^A modification related genes, just the expression level of *YTHDC2* in the S group was significantly lower than that in the R group, which is consistent with the RNA-seq results (*P* < 0.05) (**Figure 6B**). The expression level of immune related DMGs *EPG5*, *IFT74*, *SUSD5*, *STAB2* and *WDR41* in the S group were significantly lower than those in the R group, whereas the expression level of *FOS* in the S group was significantly higher than that in the R group (*P* < 0.05) (**Figure 6C**). Using MeRIP-qPCR, we found that the m^6^A modification level of *EPG5*, *STAB2* and *WDR41* in R group was increased, but the m^6^A modification levels of *SUSD5*, *IFT74* and *FOS* in R group were decreased compared to S group (*P* < 0.05) (**Figure 6D**).

**Figure 7:**
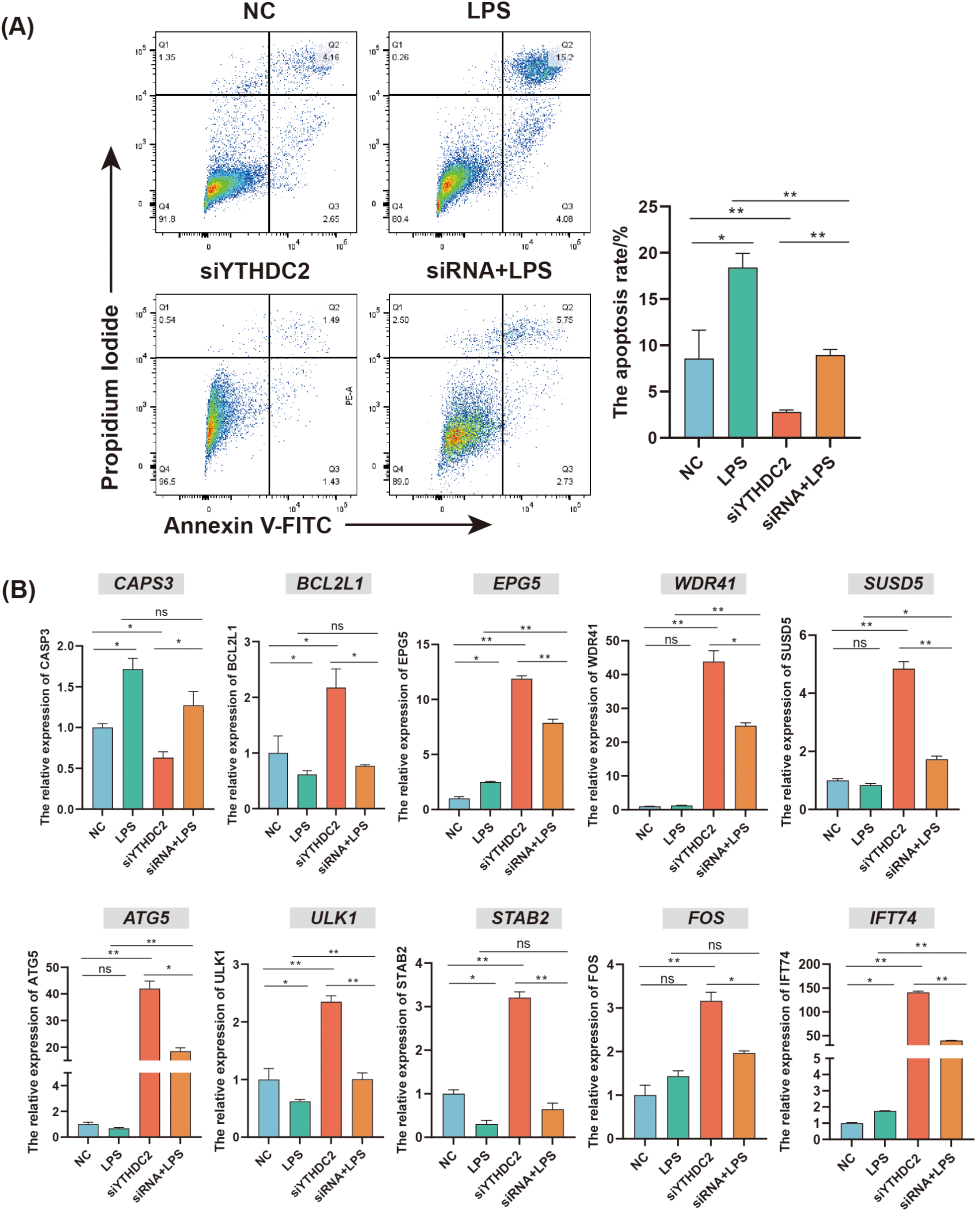
The regulatory role of YTHDC2 for chicken HD11 cell line responding to *C. jejuni* LPS stimulation. **(A)** Flow cytometric analysis of apoptosis in HD11 cells after YTHDC2 knock down followed by *C. jejuni* LPS stimulation. **(B)** Effects of interference YTHDC2 on the mRNA expression of apoptosis and autophagy related genes involved in responding to *C. jejuni* LPS stimulation. The data are pooled from 4 independent experiments with 4 replicates per group (n = 4) and presented as the mean ± SEM; * and ** represent *P* < 0.05, *P* < 0.01, respectively.

To further evaluate the function of YTHDC2, the YHTDC2 knockdown model in chicken HD11 cell line was constructed. The apoptosis rate of HD11 in LPS group was higher than that in NC group, whereas the apoptosis rate of HD11 in si-YTHDC2 group was lower than that in NC group (*P* < 0.05) (**Figure 7A**). The qRT-PCR results of apoptosis related gene *CASP3* and anti-apoptosis related gene *BCL2L1* further supported the above results (*P* < 0.05) (**Figure 7B**). Additionally, YTHDC2 knockdown could increase the expression of the autophagy related genes *ATG5* and *ULK1*, whereas LPS stimulation could decrease the expression of the *ATG5* and *ULK1* (*P* < 0.05) (**Figure 7B**). Moreover, YTHDC2 knockdown significantly increased the expression of DMGs: *EPG5*, *WDR41*, *SUSD5*, *STAB2*, *FOS*, and *IFT74* (*P* < 0.05). Compared to NC group, the expression of *EPG5* and *IFT74 was in*creased, whereas the expression of *STAB2 was* decreased in the LPS group (*P* < 0.05) (**Figure 7B**). These results indicated that YTHDC2 could be involved in regulating the apoptosis and autophagy process of HD11 cells through altering the expression of DMGs including *IFT74*, *SUSD5*, *STAB2*, *EPG5* and *FOS*.

## 4 Discussion

*C. jejuni*, as a commensal bacterium in commercial broiler chickens, seriously hampers bird welfare (49). *C. jejuni* colonizes the avian intestines in high numbers and rapidly spreads within flocks (50). Studies on pathogen-resistant and -susceptible chicken inbred lines have revealed that differences in innate immunity are associated with variations in intestinal β-defensin secretion (51). The mechanism in response to *C. jejuni* inoculation in chickens is regulated by multiple molecular levels with manifestation in transcriptional level, post-transcriptional level, and the protein level (52–54). While, the regulatory role of m^6^A modification underlying chicken responding to *C. jejuni* inoculation still remains unclear. Therefore, the chicken model inoculated with *C. jejuni* inoculation was constructed, and the landscape of m^6^A modification of chicken cecum was characterized.

m^6^A modification, the most prevalent and abundant internal post-transcriptional modification of messenger RNA in eukaryotic organisms, plays essential regulatory roles in immune responsiveness (55–58). In this study, we identified numerous m^6^A modification sites in both susceptible and resistant chickens of cecum, which were mainly located in the 3′UTR, CDS, and stop codon regions, which aligns with previously findings (59–61). m^6^A modification has been mechanistically implicated in attenuating mRNA stability and facilitating mRNA decay across diverse biological processes (62, 63). Conversely, emerging evidence suggests that m^6^A methylation density is positively correlated with transcript abundance (64–66). In our study, the expression of genes containing m^6^A peak was significantly higher than that of genes without modification, indicating the m^6^A modification could regulate chicken responding to *C. jejuni* colonization though modulating gene expression.

Recent researches have uncovered that m^6^A methylation machinery emerged as important regulators of host immunity through dynamics regulation of RNA metabolism and innate immune signaling pathways (67, 68). In the current study, we identified numerous differential m^6^A peaks associated with immune related pathways, such as regulation of canonical NF-kappaB signal transduction, apoptotic signaling pathway, MyD88-dependent toll-like receptor signaling pathway, mTOR signaling pathway. As the prototypical adapter of the Toll-like receptor signaling cascade, MyD88 coordinates essential innate immune defenses against microbial pathogens (69). Furthermore, SPOP-mediated ubiquitination limits canonical NF-κB signaling activity, thereby attenuating IL-1β biosynthesis in chicken macrophages following lipopolysaccharide challenge (70). B cell-specific mTOR deficiency can limit humoral immune responses through AID signaling (71). In the current study, the above immune related pathways were significantly enriched by DMPs *PIDD1*, *ZFAND6*, *CAPN3*, *WNT6*, *PRKCB*, *WNT4*, and *SLC38A9.* Of which, *PIDD1*, *ZFAND6*, *WNT6*, *PRKCB*, and *WNT4* were hyper-regulated in the *C. jejuni*-resistant chickens, whereas *CAPN3*, and *SLC38A9* were hypo-methylated in the *C. jejuni*-resistant chickens. These genes are involved in the immune response by inducing M1 macrophages polarization, the over-activation of innate immunity, immune infiltration, and inhibiting the proliferation of various bacteria (72–74). Dysregulation of m^6^A modification in intestinal epithelial cells could disrupt intestinal immune cell homeostasis (75). Therefore, *C. jejuni* inoculation may trigger the immune related signaling pathways by altering the methylation levels of candidate genes.

m^6^A modifications are widely acknowledged as being specifically recognized and bound by m^6^A reader proteins (76). YTHDC2 as the member of the YT521-B homology (YTH) family of proteins, contains the highly conserved YTH domain and multiple helicase domains that selectively recognizes m^6^A (77). Numerous studies have demonstrated that YTHDC2 as a crucial regulator was involved in sex differentiation (55), ferroptosis (78), Yersinia ruckeri infection (79), virus invasion (80). In the current study, we found the expression of *YTHDC2* was increased in the *C. jejuni*-susceptible chickens. Further analysis identified several DMGs *SUSD5*, *IFT74*, *WDR41*, *EPG5*, *FOS*, and *STAB2* enriched in immune related terms including Notch signaling pathway, the regulation of autophagy pathway, defense response to bacteria, and apoptosis pathway. These pathways have been validated as critical modulators of immune responses, coordinating both innate regulator functions and adaptive immune priming through transcriptional and post-translational regulation of cytokine biosynthesis and immune cell determination (81–84). Notch signaling pathway could regulate the LPS induced cellular immune and inflammatory response in chicken macrophages (85). These findings elucidate a complex regulatory mechanism of immune responses in chickens during *C. jejuni* inoculation. The autophagy-related gene *EPG5* mediates intestinal antiviral immunity through microbiota independent mechanisms (86). *IFT74* was mainly associated with intraciliary transport involved in cilium assembly (87). *STAB2* as a scavenger receptor was mainly associated with defense response to Gram-negative bacterium through inducing the production of anti-inflammatory mediators (88). FOS, a member of the AP-1 transcription factor family, plays a critical role in cell proliferation, differentiation, gene regulation, and tumorigenesis (89). Therefore, we inferred that *EPG5*, *IFT74*, *STAB2* and *SLC38A9* could involve in regulating chicken responding to *C. jejuni* colonization. *YTHDC2* can reduce the translation efficiency of target genes and the mRNA abundance in the meiosis of germline cells (77). Here, we found the knockdown of YTHDC2 could decrease the apoptosis rate of chicken HD11, and increase the expression of *IFT74*, *SUSD5*, *STAB2*, *EPG5* and *FOS*. Taken together, *YTHDC2* could regulate the apoptosis and autophagy process of HD11 cells through altering the level of m^6^A methylated modification and expression of DMGs including *IFT74*, *SUSD5*, *STAB2*, *EPG5* and *FOS* in the response to *C. jejuni* colonization.

## 5 Conclusion

In the current study, we found m^6^A methylation modification could involve in the process of chicken responding to *C. jejuni* inoculation through regulating gene expression. YTHDC2 could involve in regulating the apoptosis and autophagy process of HD11 cells through altering the expression of DMGs including *IFT74*, *SUSD5*, *STAB2*, *EPG5* and *FOS*, which was confirmed by experiments *in vitro*. This regulatory role of m^6^A methylation modification underlying chicken cecum responding to *C. jejuni* inoculation was firstly characterized. Our results would provide novel insights into understanding the molecular mechanisms underlying chicken in response to *C. jejuni* inoculation, and offer new insights for improving chicken disease resistance.

## Data Availability

The datasets presented in this study can be found in online repositories. The names of the repository/repositories and accession number(s) can be found in the article/**Supplementary Material**.

## References

[1] Tegtmeyer N Sharafutdinov I Harrer A Soltan Esmaeili D Linz B Backert S. Campylobacter virulence factors and molecular host-pathogen interactions. Curr Top Microbiol Immunol. (2021) 431:169–202. doi: 10.1007/978-3-030-65481-8_7, PMID: 33620652

[2] Awad WA Hess C Hess M. Re-thinking the chicken-Campylobacter jejuni interaction: a review. Avian Pathol. (2018) 47:352–63. doi: 10.1080/03079457.2018.1475724, PMID: 29764197

[3] Lopes GV Ramires T Kleinubing NR Scheik LK Fiorentini Â M Padilha da Silva W. Virulence factors of foodborne pathogen Campylobacterjejuni. Microb Pathog. (2021) 161:105265. doi: 10.1016/j.micpath.2021.105265, PMID: 34699927

[4] Kaakoush NO Castaño-Rodríguez N Mitchell HM Man SM. Global epidemiology of campylobacter infection. Clin Microbiol Rev. (2015) 28:687–720. doi: 10.1128/cmr.00006-15, PMID: 26062576 PMC4462680

[5] Sheppard SK Maiden MC. The evolution of Campylobacter jejuni and Campylobacter coli. Cold Spring Harb Perspect Biol. (2015) 7:a018119. doi: 10.1101/cshperspect.a018119, PMID: 26101080 PMC4526750

[6] Dessouky YE Elsayed SW Abdelsalam NA Saif NA Álvarez-Ordóñez A Elhadidy M. Genomic insights into zoonotic transmission and antimicrobial resistance in Campylobacter jejuni from farm to fork: a one health perspective. Gut Pathog. (2022) 14:44. doi: 10.1186/s13099-022-00517-w, PMID: 36471447 PMC9721040

[7] Al Hakeem WG Fathima S Shanmugasundaram R Selvaraj RK. Campylobacter jejuni in poultry: pathogenesis and control strategies. Microorganisms. (2022) 10:2134. doi: 10.3390/microorganisms10112134, PMID: 36363726 PMC9697106

[8] Knudsen KN Bang DD Andresen LO Madsen M. Campylobacter jejuni strains of human and chicken origin are invasive in chickens after oral challenge. Avian Dis. (2006) 50:10–4. doi: 10.1637/7376-051005r.1, PMID: 16617974

[9] Sanyal SC Islam KM Neogy PK Islam M Speelman P Huq MI. Campylobacter jejuni diarrhea model in infant chickens. Infect Immun. (1984) 43:931–6. doi: 10.1128/iai.43.3.931-936.1984, PMID: 6698612 PMC264273

[10] Awad WA Dublecz F Hess C Dublecz K Khayal B Aschenbach JR. Campylobacter jejuni colonization promotes the translocation of Escherichia coli to extra-intestinal organs and disturbs the short-chain fatty acids profiles in the chicken gut. Poult Sci. (2016) 95:2259–65. doi: 10.3382/ps/pew151, PMID: 27143773

[11] Omarova S Awad K Moos V Püning C Gölz G Schulzke JD. Intestinal barrier in post-campylobacter jejuni irritable bowel syndrome. Biomolecules. (2023) 13:449. doi: 10.3390/biom13030449, PMID: 36979384 PMC10046606

[12] Schoeni JL Doyle MP. Reduction of Campylobacter jejuni colonization of chicks by cecum-colonizing bacteria producing anti-C. jejuni metabolites. Appl Environ Microbiol. (1992) 58:664–70. doi: 10.1128/aem.58.2.664-670.1992, PMID: 1610187 PMC195299

[13] Shayya NW Bandick R Busmann LV Mousavi S Bereswill S Heimesaat MM. Metabolomic signatures of intestinal colonization resistance against Campylobacter jejuni in mice. Front Microbiol. (2023) 14:1331114. doi: 10.3389/fmicb.2023.1331114, PMID: 38164399 PMC10757985

[14] Wagle BR Donoghue AM Shrestha S Upadhyaya I Arsi K Gupta A. Carvacrol attenuates Campylobacter jejuni colonization factors and proteome critical for persistence in the chicken gut. Poult Sci. (2020) 99:4566–77. doi: 10.1016/j.psj.2020.06.020, PMID: 32868001 PMC7598144

[15] Pielsticker C Glünder G Rautenschlein S. Colonization properties of Campylobacter jejuni in chickens. Eur J Microbiol Immunol (Bp). (2012) 2:61–5. doi: 10.1556/EuJMI.2.2012.1.9, PMID: 24611122 PMC3933991

[16] Shaughnessy RG Meade KG Cahalane S Allan B Reiman C Callanan JJ. Innate immune gene expression differentiates the early avian intestinal response between Salmonella and Campylobacter. Vet Immunol Immunopathol. (2009) 132:191–8. doi: 10.1016/j.vetimm.2009.06.007, PMID: 19632728

[17] Psifidi A Fife M Howell J Matika O van Diemen PM Kuo R. The genomic architecture of resistance to Campylobacter jejuni intestinal colonisation in chickens. BMC Genomics. (2016) 17:293. doi: 10.1186/s12864-016-2612-7, PMID: 27090510 PMC4835825

[18] Zhang J Goto RM Psifidi A Stevens MP Taylor RL Miller MM. Research Note: MHCY haplotype impacts Campylobacter jejuni colonization in a backcross [(Line 6_1_ x Line N) x Line N] population. Poult Sci. (2022) 101:101654. doi: 10.1016/j.psj.2021.101654, PMID: 35007930 PMC8749299

[19] Connell S Meade KG Allan B Lloyd AT Downing T O’Farrelly C. Genome-wide association analysis of avian resistance to Campylobacter jejuni colonization identifies risk locus spanning the CDH13 gene. G3 (Bethesda). (2013) 3:881–90. doi: 10.1534/g3.113.006031, PMID: 23550144 PMC3656734

[20] Swaggerty CL Pevzner IY He H Genovese KJ Kogut MH. Selection for pro-inflammatory mediators produces chickens more resistant to Campylobacter jejuni. Poult Sci. (2017) 96:1623–7. doi: 10.3382/ps/pew465, PMID: 28339707

[21] Ren F Li X Tang H Jiang Q Yun X Fang L. Insights into the impact of flhF inactivation on Campylobacter jejuni colonization of chick and mice gut. BMC Microbiol. (2018) 18:149. doi: 10.1186/s12866-018-1318-1, PMID: 30348090 PMC6196472

[22] Li P Cui Y Guo F Guo J Cao X Lin J. Campylobacter jejuni infection induces dynamic expression of avian host defense peptides *in vitro* and in *vivo* . Vet Microbiol. (2023) 277:109631. doi: 10.1016/j.vetmic.2022.109631, PMID: 36543091

[23] Liu X Liu L Zhang M Wang H Yang N Li X. Chicken cecal microRNAs in the response to Campylobacter jejuni inoculation by Solexa sequencing. Poult Sci. (2016) 95:2819–23. doi: 10.3382/ps/pew190, PMID: 27303046

[24] Wang H Liu L Liu X Zhang M Li X. Correlation between miRNAs and target genes in response to Campylobacter jejuni inoculation in chicken. Poult Sci. (2018) 97:485–93. doi: 10.3382/ps/pex343, PMID: 29253230

[25] Zhao Y Wang Y Ren Y Liu L Wang T Liu L. Direct RNA sequencing reveals chicken post-transcriptional modifications in response to Campylobacter jejuni inoculation. BMC Genomics. (2025) 26:374. doi: 10.1186/s12864-025-11564-3, PMID: 40229696 PMC11998244

[26] Mao Y Dong L Liu XM Guo J Ma H Shen B. m^6^A in mRNA coding regions promotes translation via the RNA helicase-containing YTHDC2. Nat Commun. (2019) 10:5332. doi: 10.1038/s41467-019-13317-9, PMID: 31767846 PMC6877647

[27] Fan Y Zhang C Zhu G. Profiling of RNA N6-methyladenosine methylation during follicle selection in chicken ovary. Poult Sci. (2019) 98:6117–24. doi: 10.3382/ps/pez277, PMID: 31189182

[28] Chen B Liu S Zhang W Xiong T Zhou M Hu X. Profiling Analysis of N6-Methyladenosine mRNA Methylation Reveals Differential m^6^A Patterns during the Embryonic Skeletal Muscle Development of Ducks. Anim (Basel). (2022) 12:2953. doi: 10.3390/ani12192593, PMID: 36230334 PMC9559603

[29] Xu T Xu Z Lu L Zeng T Gu L Huang Y. Transcriptome-wide study revealed m^6^A regulation of embryonic muscle development in Dingan goose (Anser cygnoides orientalis). BMC Genomics. (2021) 22:270. doi: 10.1186/s12864-021-07556-8, PMID: 33853538 PMC8048326

[30] Wu L Quan W Zhang Y Wang M Ou X Mao S. Attenuated Duck Hepatitis A Virus Infection Is Associated With High mRNA Maintenance in Duckling Liver via m^6^A Modification. Front Immunol. (2022) 13:839677. doi: 10.3389/fimmu.2022.839677, PMID: 35757688 PMC9218207

[31] Zhao Q Yao Z Chen L He Y Xie Z Zhang H. Transcriptome-wide dynamics of m(6)A methylation in tumor livers induced by ALV-J infection in chickens. Front Immunol. (2022) 13:868892. doi: 10.3389/fimmu.2022.868892, PMID: 35529873 PMC9072629

[32] Hu Y Feng Y Zhang L Jia Y Cai D Qian SB. GR-mediated FTO transactivation induces lipid accumulation in hepatocytes via demethylation of m(6)A on lipogenic mRNAs. RNA Biol. (2020) 17:930–42. doi: 10.1080/15476286.2020.1736868, PMID: 32116145 PMC7549648

[33] Zhou Z Zhang A Liu X Yang Y Zhao R Jia Y. m(6)A-mediated PPARA translational suppression contributes to corticosterone-induced visceral fat deposition in chickens. Int J Mol Sci. (2022) 23:15761. doi: 10.3390/ijms232415761, PMID: 36555401 PMC9779672

[34] Wu J Frazier K Zhang J Gan Z Wang T Zhong X. Emerging role of m(6) A RNA methylation in nutritional physiology and metabolism. Obes Rev. (2020) 21:e12942. doi: 10.1111/obr.12942, PMID: 31475777 PMC7427634

[35] Yang C Hu Y Zhou B Bao Y Li Z Gong C. The role of m(6)A modification in physiology and disease. Cell Death Dis. (2020) 11:960. doi: 10.1038/s41419-020-03143-z, PMID: 33162550 PMC7649148

[36] Qiao Y Xiao G Zhu X Wen J Bu Y Zhang X. Resveratrol Enhances Antioxidant and Anti-Apoptotic Capacities in Chicken Primordial Germ Cells through m^6^A Methylation: A Preliminary Investigation. Anim (Basel). (2024) 14:2214. doi: 10.3390/ani14152214, PMID: 39123740 PMC11311097

[37] Shi H Wei J He C. Where, when, and how: context-dependent functions of RNA methylation writers, readers, and erasers. Mol Cell. (2019) 74:640–50. doi: 10.1016/j.molcel.2019.04.025, PMID: 31100245 PMC6527355

[38] Shu B Zhou YX Li H Zhang RZ He C Yang X. The METTL3/MALAT1/PTBP1/USP8/TAK1 axis promotes pyroptosis and M1 polarization of macrophages and contributes to liver fibrosis. Cell Death Discov. (2021) 7:368. doi: 10.1038/s41420-021-00756-x, PMID: 34839365 PMC8627510

[39] Zhu J Liu S Fang J Cui Z Wang B Wang Y. Enzymolysis-based RNA pull-down identifies YTHDC2 as an inhibitor of antiviral innate response. Cell Rep. (2023) 42:113192. doi: 10.1016/j.celrep.2023.113192, PMID: 37776518

[40] Li W Zhou J Gu Y Chen Y Huang Y Yang J. Lactylation of RNA m^6^A demethylase ALKBH5 promotes innate immune response to DNA herpesviruses and mpox virus. Proc Natl Acad Sci U S A. (2024) 121:e2409132121. doi: 10.1073/pnas.2409132121, PMID: 39413129 PMC11513906

[41] Chen S Zhou Y Chen Y Gu J. fastp: an ultra-fast all-in-one FASTQ preprocessor. Bioinformatics. (2018) 34:i884–90. doi: 10.1093/bioinformatics/bty560, PMID: 30423086 PMC6129281

[42] Kim D Paggi JM Park C Bennett C Salzberg SL. Graph-based genome alignment and genotyping with HISAT2 and HISAT-genotype. Nat Biotechnol. (2019) 37:907–15. doi: 10.1038/s41587-019-0201-4, PMID: 31375807 PMC7605509

[43] Meng J Lu Z Liu H Zhang L Zhang S Chen Y. A protocol for RNA methylation differential analysis with MeRIP-Seq data and exomePeak R/Bioconductor package. Methods. (2014) 69:274–81. doi: 10.1016/j.ymeth.2014.06.008, PMID: 24979058 PMC4194139

[44] Wang K Li M Hakonarson H. ANNOVAR: functional annotation of genetic variants from high-throughput sequencing data. Nucleic Acids Res. (2010) 38:e164. doi: 10.1093/nar/gkq603, PMID: 20601685 PMC2938201

[45] Heinz S Benner C Spann N Bertolino E Lin YC Laslo P. Simple combinations of lineage-determining transcription factors prime cis-regulatory elements required for macrophage and B cell identities. Mol Cell. (2010) 38:576–89. doi: 10.1016/j.molcel.2010.05.004, PMID: 20513432 PMC2898526

[46] Lee TH Kim YK Nahm BH. GBParsy: a GenBank flatfile parser library with high speed. BMC Bioinf. (2008) 9:321. doi: 10.1186/1471-2105-9-321, PMID: 18652706 PMC2516526

[47] Pertea M Pertea GM Antonescu CM Chang TC Mendell JT Salzberg SL. StringTie enables improved reconstruction of a transcriptome from RNA-seq reads. Nat Biotechnol. (2015) 33:290–5. doi: 10.1038/nbt.3122, PMID: 25690850 PMC4643835

[48] Love MI Huber W Anders S. Moderated estimation of fold change and dispersion for RNA-seq data with DESeq2. Genome Biol. (2014) 15:550. doi: 10.1186/s13059-014-0550-8, PMID: 25516281 PMC4302049

[49] Pokhrel D Thames HT Zhang L Dinh T Schilling MW White S. Aerotolerance and multi-locus sequence typing of campylobacter jejuni isolated from commercial broiler processing plants. Foods. (2023) 12:3305. doi: 10.3390/foods12173305, PMID: 37685237 PMC10486707

[50] Szczepańska B Kamiński P Andrzejewska M Śpica D Kartanas E Ulrich W. Prevalence, virulence, and antimicrobial resistance of Campylobacter jejuni and Campylobacter coli in white stork Ciconia ciconia in Poland. Foodborne Pathog Dis. (2015) 12:24–31. doi: 10.1089/fpd.2014.1793, PMID: 25456607

[51] Li X Swaggerty CL Kogut MH Chiang HI Wang Y Genovese KJ. Systemic response to Campylobacter jejuni infection by profiling gene transcription in the spleens of two genetic lines of chickens. Immunogenetics. (2012) 64:59–69. doi: 10.1007/s00251-011-0557-1, PMID: 21748442

[52] Ruddell B Hassall A Sahin O Plummer PJ Zhang Q Kreuder AJ. Small RNA CjNC110 regulates the activated methyl cycle to enable optimal chicken colonization by Campylobacter jejuni. mSphere. (2025) 10:e0083224. doi: 10.1128/msphere.00832-24, PMID: 39772717 PMC11774046

[53] Russell KM Smith J Bremner A Chintoan-Uta C Vervelde L Psifidi A. Transcriptomic analysis of caecal tissue in inbred chicken lines that exhibit heritable differences in resistance to Campylobacter jejuni. BMC Genomics. (2021) 22:411. doi: 10.1186/s12864-021-07748-2, PMID: 34082718 PMC8176612

[54] Shank JM Kelley BR Jackson JW Tweedie JL Franklin D Damo SM. The Host Antimicrobial Protein Calgranulin C Participates in the Control of Campylobacter jejuni Growth via Zinc Sequestration. Infect Immun. (2018) 86:e00234-18. doi: 10.1128/iai.00234-18, PMID: 29610259 PMC5964530

[55] Li J Zhang X Wang X Sun C Zheng J Li J. The m^6^A methylation regulates gonadal sex differentiation in chicken embryo. J Anim Sci Biotechnol. (2022) 13:52. doi: 10.1186/s40104-022-00710-6, PMID: 35581635 PMC9115958

[56] Wang Z Ju X Li K Cai D Zhou Z Nie Q. MeRIP sequencing reveals the regulation of N6-methyladenosine in muscle development between hypertrophic and leaner broilers. Poult Sci. (2024) 103:103708. doi: 10.1016/j.psj.2024.103708, PMID: 38631230 PMC11040168

[57] Yu B Liu J Cai Z Wang H Feng X Zhang T. RNA N(6)-methyladenosine profiling reveals differentially methylated genes associated with intramuscular fat metabolism during breast muscle development in chicken. Poult Sci. (2023) 102:102793. doi: 10.1016/j.psj.2023.102793, PMID: 37276703 PMC10258505

[58] Zhao Y Jiang Y Feng Y Zhao R. RNA m(6)A-mediated post-transcriptional repression of glucocorticoid receptor in LPS-activated Kupffer cells on broilers. Poult Sci. (2025) 104:104393. doi: 10.1016/j.psj.2024.104393, PMID: 39571201 PMC11617446

[59] Luo G Wang S Ai Y Li J Ren Z. N6-methyladenosine methylome profiling of muscle and adipose tissues reveals methylase-mRNA metabolic regulatory networks in fat deposition of rex rabbits. Biol (Basel). (2022) 11:944. doi: 10.3390/biology11070944, PMID: 36101325 PMC9312354

[60] Ma X La Y Bao P Chu M Guo X Wu X. Regulatory role of N6-methyladenosine in longissimus dorsi development in yak. Front Vet Sci. (2022) 9:757115. doi: 10.3389/fvets.2022.757115, PMID: 35498742 PMC9043854

[61] Meyer KD Saletore Y Zumbo P Elemento O Mason CE Jaffrey SR. Comprehensive analysis of mRNA methylation reveals enrichment in 3’ UTRs and near stop codons. Cell. (2012) 149:1635–46. doi: 10.1016/j.cell.2012.05.003, PMID: 22608085 PMC3383396

[62] Wang X Lu Z Gomez A Hon GC Yue Y Han D. N6-methyladenosine-dependent regulation of messenger RNA stability. Nature. (2014) 505:117–20. doi: 10.1038/nature12730, PMID: 24284625 PMC3877715

[63] Zhao BS Roundtree IA He C. Post-transcriptional gene regulation by mRNA modifications. Nat Rev Mol Cell Biol. (2017) 18:31–42. doi: 10.1038/nrm.2016.132, PMID: 27808276 PMC5167638

[64] Liu L Wang J Sun G Wu Q Ma J Zhang X. m(6)A mRNA methylation regulates CTNNB1 to promote the proliferation of hepatoblastoma. Mol Cancer. (2019) 18:188. doi: 10.1186/s12943-019-1119-7, PMID: 31870368 PMC6927193

[65] Wang YN Jin HZ. Transcriptome-wide m(6)A methylation in skin lesions from patients with psoriasis vulgaris. Front Cell Dev Biol. (2020) 8:591629. doi: 10.3389/fcell.2020.591629, PMID: 33251217 PMC7674922

[66] Yue B Song C Yang L Cui R Cheng X Zhang Z. METTL3-mediated N6-methyladenosine modification is critical for epithelial-mesenchymal transition and metastasis of gastric cancer. Mol Cancer. (2019) 18:142. doi: 10.1186/s12943-019-1065-4, PMID: 31607270 PMC6790244

[67] Lin C Zeng M Song J Li H Feng Z Li K. PRRSV alters m(6)A methylation and alternative splicing to regulate immune, extracellular matrix-associated function. Int J Biol Macromol. (2023) 253:126741. doi: 10.1016/j.ijbiomac.2023.126741, PMID: 37696370

[68] Luo S Liao C Zhang L Ling C Zhang X Xie P. METTL3-mediated m^6^A mRNA methylation regulates neutrophil activation through targeting TLR4 signaling. Cell Rep. (2023) 42:112259. doi: 10.1016/j.celrep.2023.112259, PMID: 36920907

[69] Gurung P Fan G Lukens JR Vogel P Tonks NK Kanneganti TD. Tyrosine kinase SYK licenses myD88 adaptor protein to instigate IL-1α-mediated inflammatory disease. Immunity. (2017) 46:635–48. doi: 10.1016/j.immuni.2017.03.014, PMID: 28410990 PMC5501252

[70] Li Q Wang F Wang Q Zhang N Zheng J Zheng M. SPOP promotes ubiquitination and degradation of MyD88 to suppress the innate immune response. PloS Pathog. (2020) 16:e1008188. doi: 10.1371/journal.ppat.1008188, PMID: 32365080 PMC7224567

[71] Zhang S Pruitt M Tran D Du Bois W Zhang K Patel R. B cell-specific deficiencies in mTOR limit humoral immune responses. J Immunol. (2013) 191:1692–703. doi: 10.4049/jimmunol.1201767, PMID: 23858034 PMC3906844

[72] Chen Y Huang D Xie A Shan Y Zhao S Gao C. Capn3b-deficient zebrafish model reveals a key role of autoimmune response in LGMDR1. J Genet Genomics. (2024) 51:1375–88. doi: 10.1016/j.jgg.2024.09.011, PMID: 39349278

[73] Garcia-Carpio I Braun VZ Weiler ES Leone M Niñerola S Barco A. Extra centrosomes induce PIDD1-mediated inflammation and immunosurveillance. EMBO J. (2023) 42:e113510. doi: 10.15252/embj.2023113510, PMID: 37530438 PMC10577638

[74] Wang J Shi M Zhang H Zhou H Huang Z Zhou Y. PRKCB is relevant to prognosis of lung adenocarcinoma through methylation and immune infiltration. Thorac Cancer. (2022) 13:1837–49. doi: 10.1111/1759-7714.14466, PMID: 35567329 PMC9200888

[75] Ma Y Zhang X Xuan B Li D Yin N Ning L. Disruption of CerS6-mediated sphingolipid metabolism by FTO deficiency aggravates ulcerative colitis. Gut. (2024) 73:268–81. doi: 10.1136/gutjnl-2023-330009, PMID: 37734910

[76] Xiao W Adhikari S Dahal U Chen YS Hao YJ Sun BF. Nuclear m(6)A Reader YTHDC1 Regulates mRNA Splicing. Mol Cell. (2016) 61:507–19. doi: 10.1016/j.molcel.2016.01.012, PMID: 26876937

[77] Hsu PJ Zhu Y Ma H Guo Y Shi X Liu Y. Ythdc2 is an N(6)-methyladenosine binding protein that regulates mammalian spermatogenesis. Cell Res. (2017) 27:1115–27. doi: 10.1038/cr.2017.99, PMID: 28809393 PMC5587856

[78] Li Y Guo M Qiu Y Li M Wu Y Shen M. Autophagy activation is required for N6-methyladenosine modification to regulate ferroptosis in hepatocellular carcinoma. Redox Biol. (2024) 69:102971. doi: 10.1016/j.redox.2023.102971, PMID: 38056309 PMC10749285

[79] Yu H Gao Q Wang W Liu D He J Tian Y. Comprehensive Analysis of YTH Domain-Containing Genes, Encoding m(6)A Reader and Their Response to Temperature Stresses and Yersinia ruckeri Infection in Rainbow Trout (Oncorhynchus mykiss). Int J Mol Sci. (2023) 24:9348. doi: 10.3390/ijms24119348, PMID: 37298300 PMC10253868

[80] Macveigh-Fierro D Cicerchia A Cadorette A Sharma V Muller M. The m(6)A reader YTHDC2 is essential for escape from KSHV SOX-induced RNA decay. Proc Natl Acad Sci U S A. (2022) 119:e2116662119. doi: 10.1073/pnas.2116662119, PMID: 35177478 PMC8872733

[81] Qin C Lu Y Bai L Wang K. The molecular regulation of autophagy in antimicrobial immunity. J Mol Cell Biol. (2022) 14:mjac015. doi: 10.1093/jmcb/mjac015, PMID: 35278083 PMC9335221

[82] Sun H Bi R Liu P Nolan LK Lamont SJ. Combined analysis of primary lymphoid tissues’ transcriptomic response to extra-intestinal Escherichia coli (ExPEC) infection. Dev Comp Immunol. (2016) 57:99–106. doi: 10.1016/j.dci.2015.12.013, PMID: 26710679

[83] Sun H Yang Y Cao Y Li H Qu L Lamont SJ. Gene expression profiling of RIP2-knockdown in HD11 macrophages - elucidation of potential pathways (gene network) when challenged with avian pathogenic E.coli (APEC). BMC Genomics. (2022) 23:341. doi: 10.1186/s12864-022-08595-5, PMID: 35501708 PMC9063279

[84] Zhang B Hong L Ke J Zhong Y Cao N Li W. Polysaccharide of Atractylodes macrocephala Koidz alleviate lipopolysaccharide-induced liver injury in goslings via the p53 and FOXO pathways. Poult Sci. (2023) 102:102480. doi: 10.1016/j.psj.2023.102480, PMID: 36680857 PMC9871332

[85] Tan J Lu Y Li H Sun H Han W Zhang J. Functional analysis of circSTX8 in chicken macrophages under lipopolysaccharide stimulation. Res Vet Sci. (2023) 165:105053. doi: 10.1016/j.rvsc.2023.105053, PMID: 37856945

[86] Lee S Kalugotla G Ingle H Rodgers R Wu C Wang Y. Intestinal antiviral signaling is controlled by autophagy gene Epg5 independent of the microbiota. Autophagy. (2022) 18:1062–77. doi: 10.1080/15548627.2021.1968607, PMID: 34520306 PMC9196718

[87] Bakey Z Cabrera OA Hoefele J Antony D Wu K Stuck MW. IFT74 variants cause skeletal ciliopathy and motile cilia defects in mice and humans. PloS Genet. (2023) 19:e1010796. doi: 10.1371/journal.pgen.1010796, PMID: 37315079 PMC10298753

[88] Penberthy KK Ravichandran KS. Apoptotic cell recognition receptors and scavenger receptors. Immunol Rev. (2016) 269:44–59. doi: 10.1111/imr.12376, PMID: 26683144 PMC4685734

[89] Li C Wang Q Peng Z Lin Y Liu H Yang X. Generation of FOS gene knockout lines from a human embryonic stem cell line using CRISPR/Cas9. Stem Cell Res. (2019) 39:101479. doi: 10.1016/j.scr.2019.101479, PMID: 31229900

