# Supplementary material for: Transcriptome-wide N6-methyladenosinem modifications analysis of chicken cecum in responding to Campylobacter jejuni inoculation
Source: Front Immunol. 2025 Jul 30;16:1630008. doi: 10.3389/fimmu.2025.1630008 (PMC12343223; doi:10.3389/fimmu.2025.1630008)
Supplement: Supplementary file 1 [file SupplementaryFile1.zip › Supplementary Figures.DOCX]

Supplementary Material

# Supplementary Figures and Tables

## Supplementary Figures


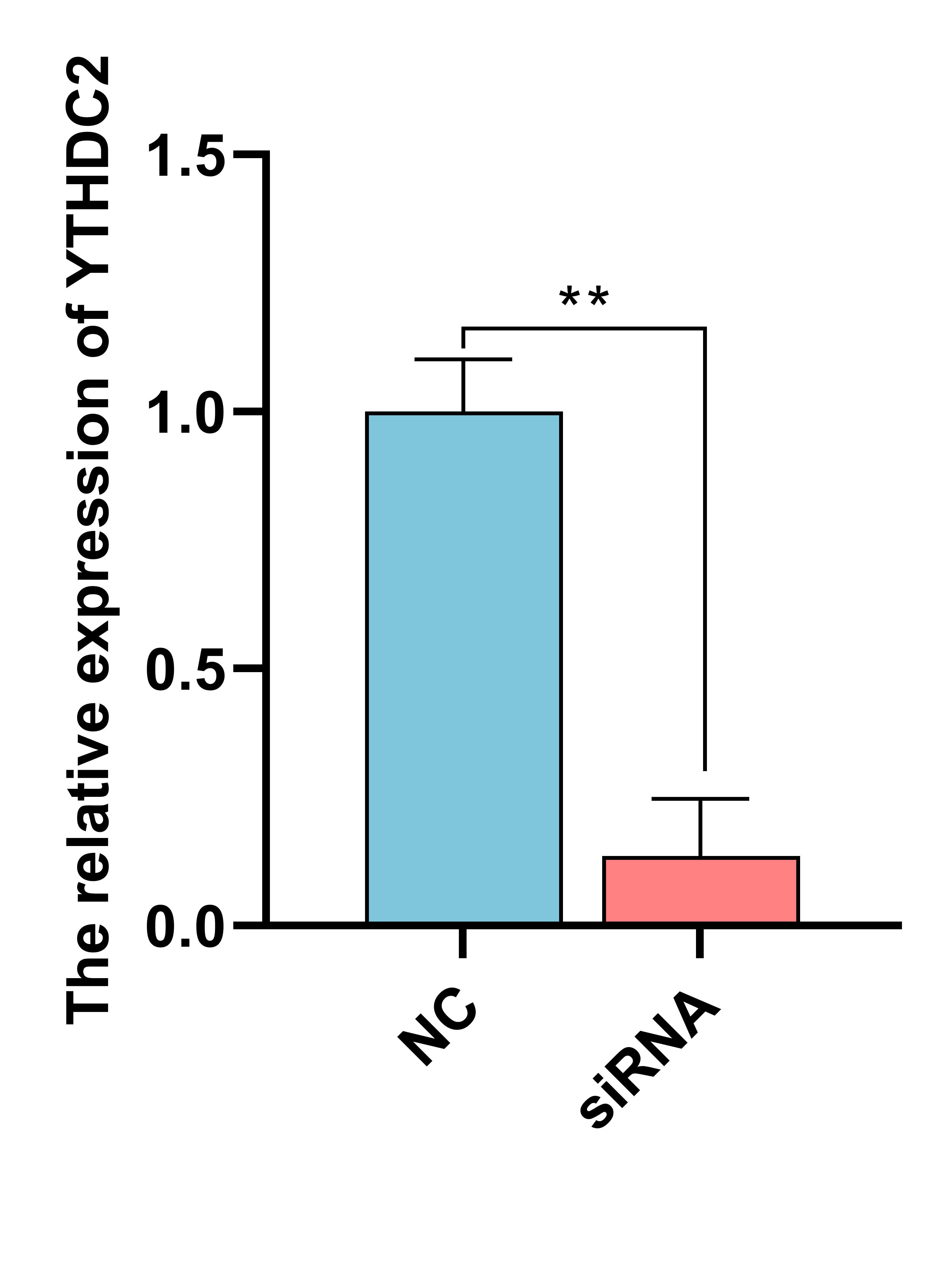


**Supplementary Figure 1.** The knockdown efficiency of YTHDC2 in chicken HD11 cell lines.


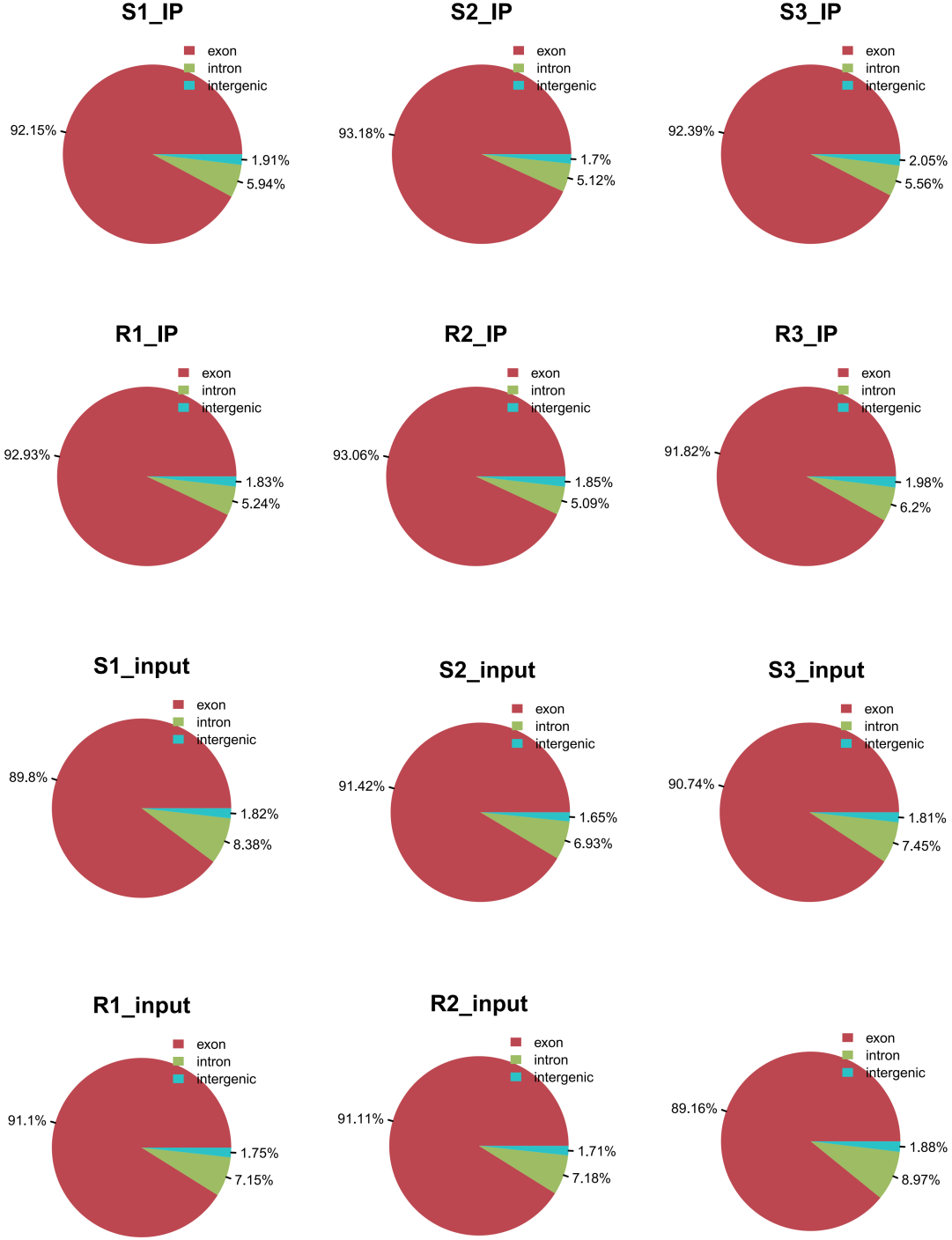


**Supplementary Figure 2.** Regional distribution of valid reads in in the resistant and susceptible groups compared to the reference genome.


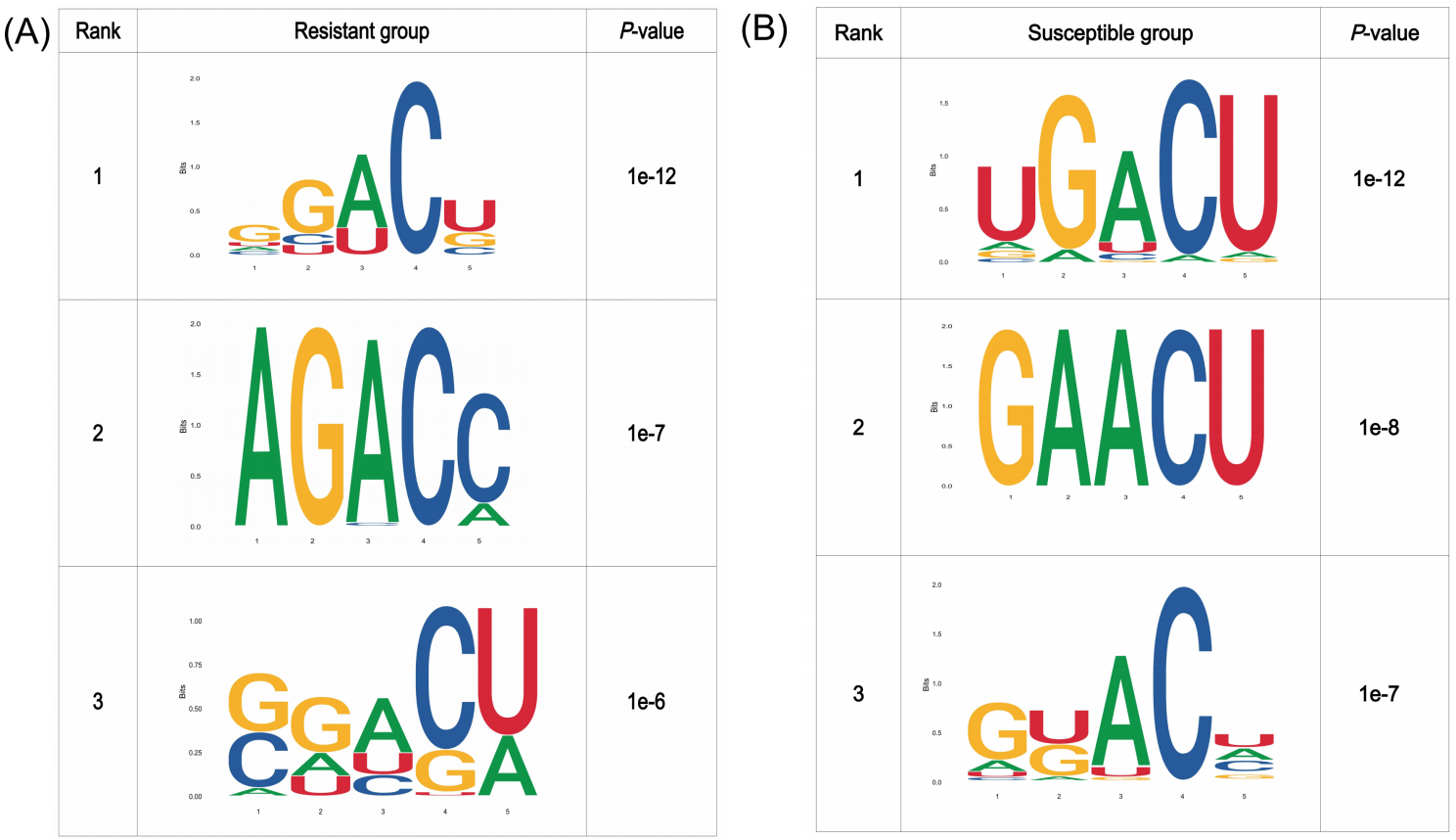


**Supplementary Figure 3.** The top motifs of m^6^A peaks in the resistant and susceptible groups. (A) The top motifs of m^6^A peaks in the resistant group. (B) The top motifs of m^6^A peaks in the susceptible group.


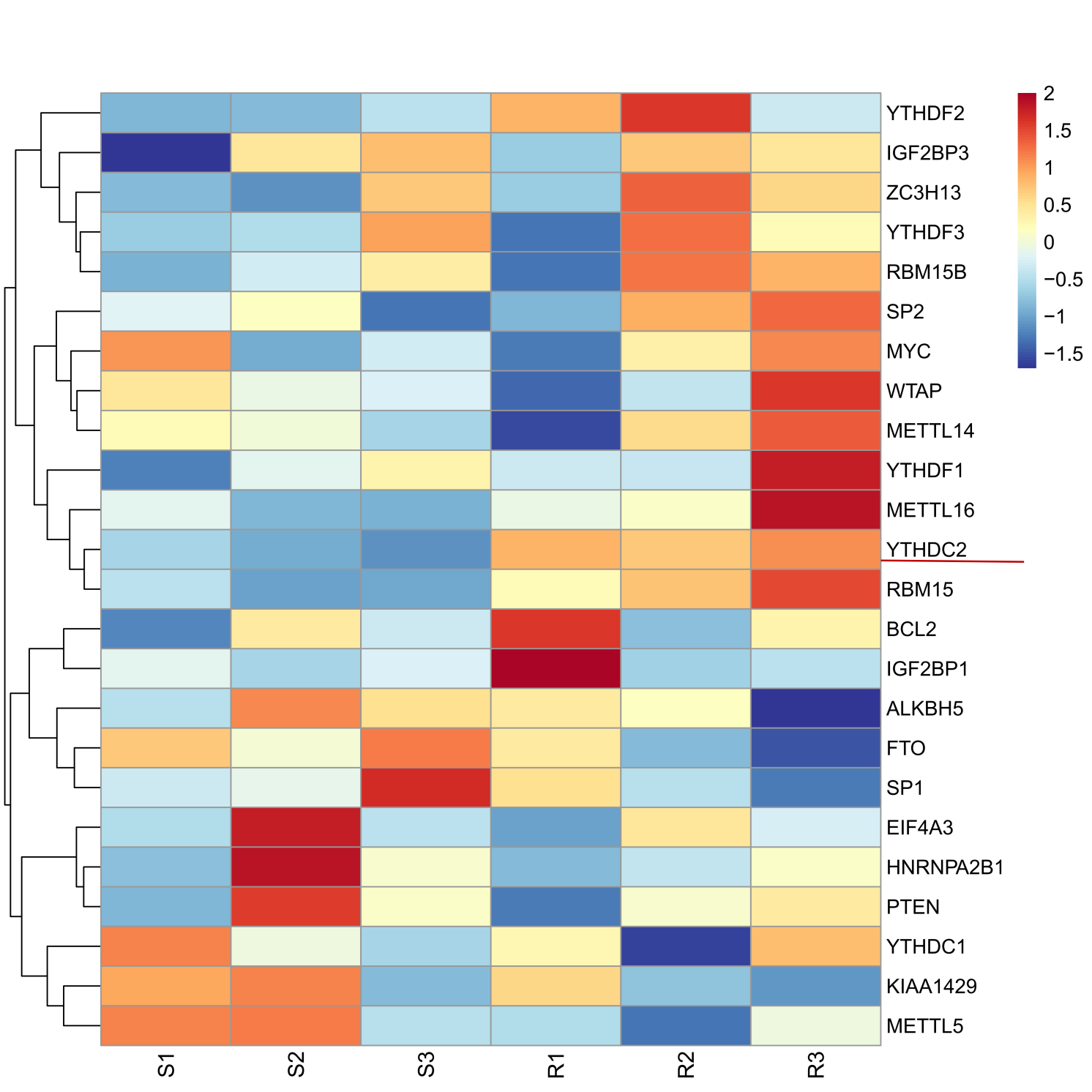


**Supplementary Figure 4.** Heat map of clustering for m^6^A modification related genes between resistant and susceptible groups.
